# NINL and DZANK1 Co-function in Vesicle Transport and Are Essential for Photoreceptor Development in Zebrafish

**DOI:** 10.1371/journal.pgen.1005574

**Published:** 2015-10-20

**Authors:** Margo Dona, Ruxandra Bachmann-Gagescu, Yves Texier, Grischa Toedt, Lisette Hetterschijt, Edith L. Tonnaer, Theo A. Peters, Sylvia E. C. van Beersum, Judith G. M. Bergboer, Nicola Horn, Erik de Vrieze, Ralph W. N. Slijkerman, Jeroen van Reeuwijk, Gert Flik, Jan E. Keunen, Marius Ueffing, Toby J. Gibson, Ronald Roepman, Karsten Boldt, Hannie Kremer, Erwin van Wijk

**Affiliations:** 1 Department of Otorhinolaryngology, Radboud University Medical Centre, Nijmegen, the Netherlands; 2 Radboud Institute for Molecular Life Sciences, Radboud University Nijmegen, Nijmegen, the Netherlands; 3 Institute of Molecular Life Sciences, University of Zurich, Zürich, Switzerland; 4 Institute of Medical Genetics, University of Zurich, Zürich, Switzerland; 5 Division of Experimental Ophthalmology, and Medical Proteome Center, Centre for Ophthalmology, Eberhard Karls University Tuebingen, Tübingen, Germany; 6 Structural and Computational Biology Unit, European Molecular Biology Laboratory, Heidelberg, Germany; 7 Department of Human Genetics, Radboud University Medical Centre, Nijmegen, the Netherlands; 8 Department of Organismal Animal Physiology, Institute for Water and Wetland Research, Radboud University Nijmegen, Nijmegen, The Netherlands; 9 Department of Ophthalmology, Radboud University Medical Centre, Nijmegen, the Netherlands; Washington University School of Medicine, UNITED STATES

## Abstract

Ciliopathies are Mendelian disorders caused by dysfunction of cilia, ubiquitous organelles involved in fluid propulsion (motile cilia) or signal transduction (primary cilia). Retinal dystrophy is a common phenotypic characteristic of ciliopathies since photoreceptor outer segments are specialized primary cilia. These ciliary structures heavily rely on intracellular minus-end directed transport of cargo, mediated at least in part by the cytoplasmic dynein 1 motor complex, for their formation, maintenance and function. Ninein-like protein (NINL) is known to associate with this motor complex and is an important interaction partner of the ciliopathy-associated proteins lebercilin, USH2A and CC2D2A. Here, we scrutinize the function of NINL with combined proteomic and zebrafish *in vivo* approaches. We identify Double Zinc Ribbon and Ankyrin Repeat domains 1 (DZANK1) as a novel interaction partner of NINL and show that loss of Ninl, Dzank1 or both synergistically leads to dysmorphic photoreceptor outer segments, accumulation of trans-Golgi-derived vesicles and mislocalization of Rhodopsin and Ush2a in zebrafish. In addition, retrograde melanosome transport is severely impaired in zebrafish lacking Ninl or Dzank1. We further demonstrate that NINL and DZANK1 are essential for intracellular dynein-based transport by associating with complementary subunits of the cytoplasmic dynein 1 motor complex, thus shedding light on the structure and stoichiometry of this important motor complex. Altogether, our results support a model in which the NINL-DZANK1 protein module is involved in the proper assembly and folding of the cytoplasmic dynein 1 motor complex in photoreceptor cells, a process essential for outer segment formation and function.

## Author Summary

The cytoplasmic dynein 1 motor complex is known to be essential for photoreceptor outer segment formation and function. NINL, an important interaction partner of three ciliopathy-associated proteins (lebercilin, USH2A and CC2D2A), was previously shown to associate with this motor complex. In this work, we scrutinize the role of NINL using a combination of affinity proteomics and zebrafish studies, in order to gain insight into the pathogenic mechanisms underlying these three associated hereditary disorders. We identify DZANK1 as an important interaction partner of NINL and show that loss of Ninl, Dzank1, or a combination of both synergistically results in impaired transport of trans Golgi-derived vesicles and, as a consequence, defective photoreceptor outer segment formation. Using affinity proteomics, we demonstrate that NINL and DZANK1 associate with complementary subunits of the cytoplasmic dynein 1 complex. Our results support a model in which the NINL-DZANK1 protein module is essential for the proper assembly and folding of the cytoplasmic dynein 1 motor complex, shedding light on the structure and stoichiometry of this important motor complex.

## Introduction

Dysfunction of cilia is the underlying defect in a growing group of pleiotropic genetic disorders, the ciliopathies. Cilia are ubiquitous microtubule-based organelles involved in fluid propulsion (motile cilia) or signal transduction (primary cilia) and ciliopathy-associated proteins localize to various ciliary sub-compartments. Retinal dystrophy is a common clinical feature of ciliopathies where the primary affected retinal cell type is the photoreceptor, which contains a highly specialized primary cilium, consisting of the connecting cilium and axoneme serving as a backbone to the outer segment. For propagation of visual excitation, outer segments are composed of stacks of membranous discs, which are densely packed with the light-sensitive transmembrane receptor rhodopsin and its associated photo-transduction machinery. The membranous disks are organized around the axoneme that is continuous with the connecting cilium. The entire outer segment can thus be regarded as a highly specialized primary cilia compartment. The connecting cilium literally connects the outer segment to the inner segment of the photoreceptor and is the equivalent of a canonical ciliary transition zone. This proximal region of the cilium ensures a tight control of protein access into the ciliary compartment [1–5] through a gate-keeper function, involving several ciliopathy-associated proteins such as NPHP’s [4] and Meckel and Joubert syndrome proteins [6], importins and Ran GTPases [7, 8].

Given the daily renewal of about 10% of the total length of the outer segments in humans [9], photoreceptor cells require intense intracellular trafficking to build their outer segments and to replenish the shed discs. Transmembrane proteins, such as rhodopsin and Usherin are synthesized in the inner segment and subsequently moved from the trans-Golgi network (TGN) towards the base of the ciliary compartment via microtubule-based vesicular transport [10]. This transport involves motor proteins such as the ATPases kinesin and dynein [11, 12]. Specifically, the cytoplasmic dynein 1 motor complex, which consists of two 530 kDa heavy chains, responsible for force production, a group of 74 kDa intermediate chains, 53 to 57 kDa light intermediate chains, and 8 to 21 kDa light chains [13], has been implicated in minus-end directed transport of post-Golgi-derived rhodopsin-containing vesicles [14]. During its transport, the carboxy-terminal domain of rhodopsin binds to the dynein light chain Tctex-type DYNLT1 [14]. In the absence of rhodopsin, small rudimentary photoreceptor outer segments are formed during the first few postnatal weeks. After this period the outer segments vanish and photoreceptors die rapidly. As a consequence, photo-transduction is impaired leading to defects in visual function [15, 16]. A similar defect in photoreceptor morphology and function is observed in the zebrafish *cannonball* mutant, in which the cytoplasmic dynein motor complex 1 is dysfunctional due to a mutation in the dynein cytoplasmic 1 heavy chain 1 (*dync1h1*) gene [17]. These findings emphasize the importance of the cytoplasmic dynein motor complex 1 for intracellular trafficking which is essential for photoreceptor development and function. However, the structure of this complex has not been fully elucidated to date.

Previously, we described a retinal ciliopathy-associated protein module consisting of Usherin, Lebercilin (Leber’s congenital amaurosis type 5) and NINL^isoB^ (ninein-like protein isoform B) present at the base of the connecting cilium [18]. In addition, we now identified a physical interaction between NINL^isoB^ and the ciliopathy protein CC2D2A, involved in Joubert and Meckel syndrome, two important ciliopathies (Bachmann-Gagescu, *et al* co-submission). Furthermore, NINL was found to associate with several members of the cytoplasmic dynein 1-dynactin motor complex and polo-like kinase 1 and was found to function in microtubule nucleation by recruitment of gamma-tubulin ring complexes [19]. However, the function of NINL in photoreceptors is still elusive.

In the current study, we investigate the role of NINL and its associated protein complex in the retina using a combination of proteomics and *in vivo* studies in zebrafish. We identify a central role for NINL and its novel interaction partner DZANK1 in vesicle transport towards the photoreceptor outer segments. Knockdown in zebrafish larvae of either *ninl* or d*zank1* or synergistically at sub-phenotypic doses, leads to abnormal outer segment morphology, mislocalization of rhodopsin, accumulation of vesicular structures and consequently, loss of visual function. We further demonstrate that NINL and DZANK1 associate with complementary subunits of the cytoplasmic dynein 1 motor complex, which is essential for proper transport of vesicle-bound proteins towards the base of the photoreceptor connecting cilium and, as a consequence, photoreceptor development in zebrafish. Together, our findings shed light onto the assembly and structure of the cytoplasmic dynein 1 motor complex and link it to several ciliopathy proteins located at the entrance to the ciliary compartment.

## Results

### DZANK1 interacts with NINL^isoB^ in the retina

**Fig 1:**
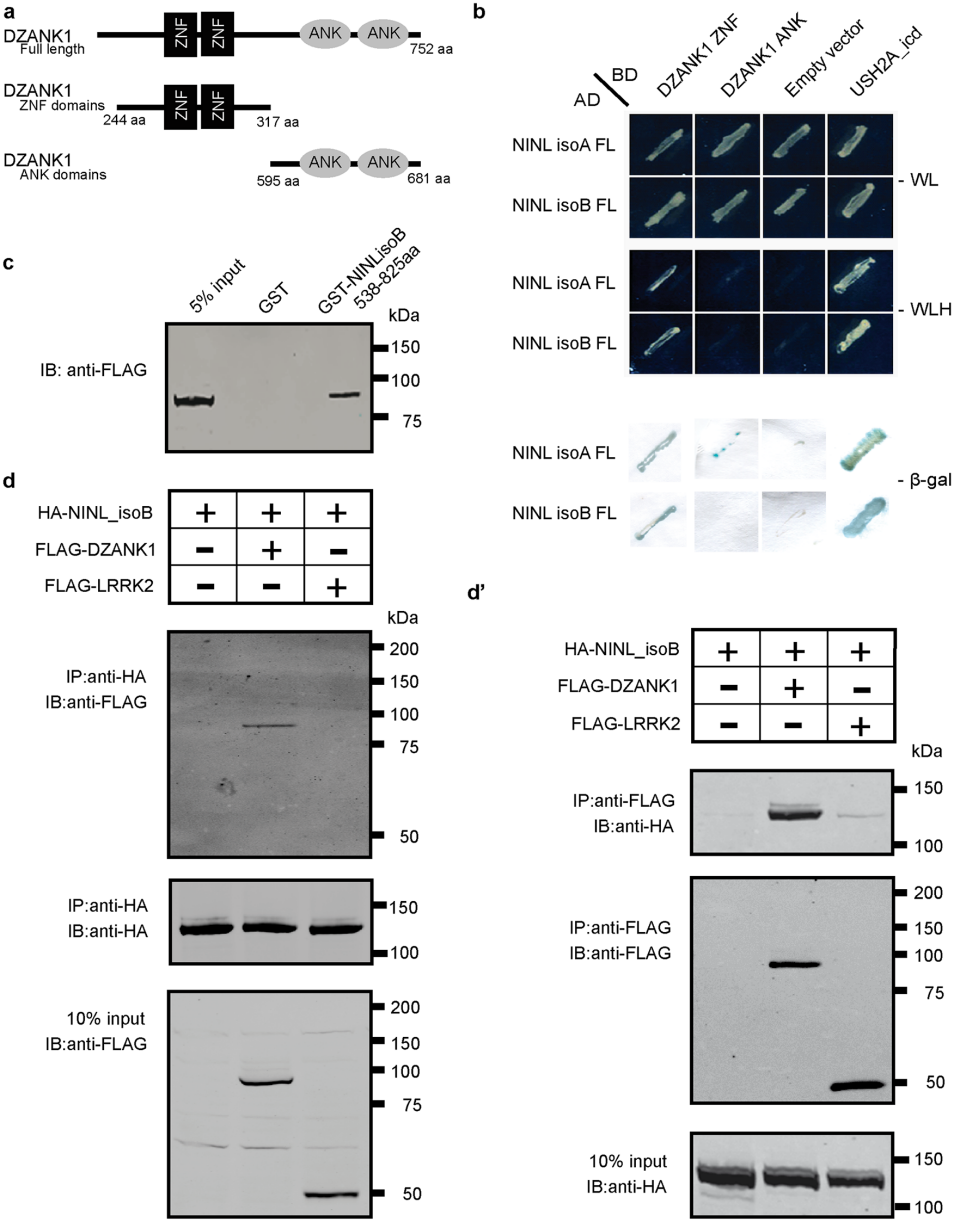
Protein-protein interaction studies. (a) Schematic protein structure of DZANK1. Two different deletion constructs of DZANK1, one consisting of the ZNF_RBZ domains (244-317aa) and the other of the ANK domains (595-681aa), were used for co-transformation in yeast. (b) Results of the co-transformation in yeast. Yeast transformants were selected on low (SD/-Trp/-Leu/-His) and high-stringency (SD/-Trp/-Leu/-His-/-Ade) medium with observed growth, indicating interaction of the tested bait and prey proteins. In addition, the β-galactosidase filter lift assay was performed. The USH2A_intracellular region was used as a positive control. Empty prey vector was used as a negative control. Yeast-two-hybrid analysis revealed a specific interaction between DZANK1 ZNF_RBZ domains and both NINL^isoA/B^. (c) GST pull-down assays, showing that Strep/FLAG-tagged DZANK1 was efficiently pulled down by GST-fused NINL^isoB^, but not by GST alone. The first lane shows 5% input of the protein lysate. (d) Co-immunoprecipitation of DZANK1 Full Length (FL) with NINL^isoB^, but not with LRRK2. The immunoblot (IB) in the top panel shows that HA-tagged NINL co-immunoprecipitated with Strep/FLAG-tagged DZANK1 (lane 2), whereas unrelated FLAG-tagged LRRK2 (lane 3) did not. The anti-HA immunoprecipitates are shown in the middle panel; protein input is shown in the bottom panel. (d’) Reciprocal IP experiments using anti-FLAG antibodies confirmed the co-immunoprecipitation of HA-tagged NINL^isoB^ with Strep/FLAG-tagged DZANK1 (lane 2) and not with LRRK2 (lane 3) shown in the top panel. The anti-FLAG immunoprecipitations are shown in the middle panel; protein input is shown in the bottom panel. ANK: ankyrin repeat domain; ZNF_RBZ: Zinc Finger domain like in Ran-binding proteins; aa: amino acids.

**Fig 2:**
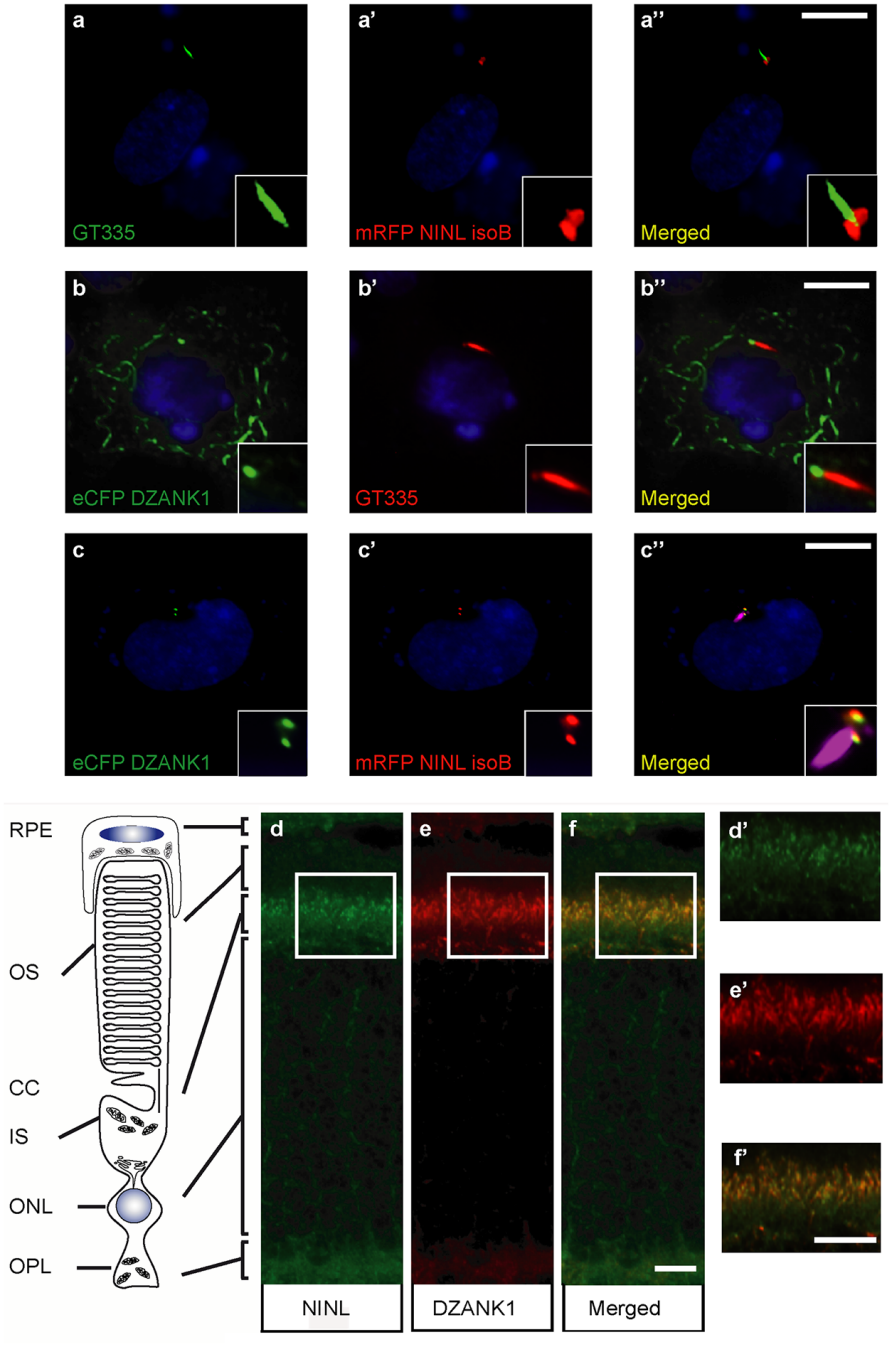
NINL and DZANK1 co-localize at the base of cilia in RPE cells and rat retina. (a-c) Centrosomal co-localization of NINL^isoB^ and DZANK1 in hTERT-RPE1 cells. When expressed alone, mRFP-NINL^isoB^ (red signal in, a-a” and c-c”) was localized to both centrioles at the base of the cilia marked with GT335 (green signal in a’-a” and red signal in b-b”), whereas eYFP–DZANK1 was localized to the basal body and to the microtubule network of the cell (green signal in b-b”). After co-expression of NINL^isoB^ and DZANK1, both proteins were localized at the basal body of the cilia in the centrosome (cilia marked by GT335, cyanid signal), supporting an interaction between the two proteins (c-c”). Nuclei were stained with DAPI (blue signal). (d-f) Co-localization of endogenous NINL^isoB^ and DZANK1 in rat retina. (d-e) Co-immunostaining of NINL and DZANK1 in radial cryo-sections of adult (P20) rat retina with anti-NINL antibodies (green signal; d) and anti-DZANK1 antibodies (red signal; e) showing co-localization (yellow signal: f) in the inner segment (IS) and in the region of the connecting cilium (CC). (d’-f’) show details of the subcellular (co-) localization of NINL and DZANK1 in, respectively, d, e and f. Scale bars represent 10 μm (a-c”), 5 μm (f) and 1 μm (f’).

Previously, NINL^isoB^ was identified as a key connector of three large retinal ciliopathies [18] (Bachmann-Gagescu *et al*, co-submission). Its function within the retina, however, has not yet been deciphered. To get insight into the function of NINL, we screened a random-primed bovine retina cDNA library to identify interaction partners for the previously predicted intermediate filament (IF) domain (538-825aa) of NINL^isoB^ [18]. Approximately 70% of the positive clones (> 1,000 clones), could be identified as DZANK1 (double zinc ribbon and ankyrin repeat domains 1), a protein with a yet unknown function. To elaborate on this finding, three overlapping cDNA fragments were cloned: one encoding full length DZANK1 (752aa) and two different deletion constructs, encoding either the zinc finger domains as found in Ran binding proteins (ZNF_RBZ, SMART accession number SM00547, 244-317aa) or the ankyrin repeats known to be involved in protein-protein interactions (ANK, SMART accession number SM00248, 595-681aa) (SMART database; http://smart.embl-heidelberg.de) (Fig 1a). To test whether the interactions of DZANK1 with NINL are isoform-specific, both isoforms of NINL were tested. The transcript encoding NINL^isoB^ lacks exon17 of the originally described *NINL* gene (encoding NINL^isoA^), resulting in the in-frame skipping of 349 amino acids after residue 734 [18]. Using dedicated binary yeast two-hybrid assays, we were able to pinpoint the interaction to the ZNF_RBZ domains of DZANK1 and both isoforms of NINL (Fig 1b). Since NINL^isoB^ was originally found in the yeast-two hybrid screen, we continued with this isoform for further confirmations. We performed several *in vitro* and *in vivo* binding assays to conclusively validate the interaction between DZANK1 and NINL^isoB^. In a glutathione S-transferase (GST) pull-down assay, we identified that full-length Strep/FLAG-tagged DZANK1 was pulled down from HEK293T cell lysates by GST-fused NINL^isoB^_538-825aa and not by GST alone (Fig 1c). To confirm this interaction *in cellulo*, HEK293T cells were co-transfected with plasmids encoding full length HA-tagged NINL^isoB^ and Strep/FLAG-tagged DZANK1 for immunoprecipitation (IP) assays. With anti-HA antibodies, NINL^isoB^ consistently co-immunoprecipitated with full length DZANK1, but not with the control protein LRRK2 (Fig 1d). Reciprocal IP experiments with anti-FLAG antibodies for IP confirmed these results (Fig 1d’). In addition we performed GST pull-down assays from biologically relevant bovine retinal extracts followed by mass spectrometry (LC-MS/MS) analysis. In total, 445 different proteins were identified of which 59 passed the stringent filtering criteria. GST-fused human NINL^isoB^_aa538-825 was able to consistently pull-down endogenous bovine DZANK1 (in 4 out of 4 assays) whereas unfused GST (in 0 out of 3 assays) was not, which further confirmed the interaction (S1 Table). Further confirmation of the interaction was obtained through co-localization of eGFP- and mRFP-tagged DZANK1 and NINL^isoB^ in hTERT-RPE1 cells. In singly transfected cells, NINL^isoB^ and DZANK1 were both present at the ciliary base, partially co-localizing with the basal body and the ciliary marker polyglutamylated tubulin (anti-GT335) (Fig 2a and 2b”). DZANK1 also localized along the microtubule network of the cells. Co-expression of NINL^isoB^ and DZANK1 resulted in the co-localization of both proteins, thereby fully retaining the latter to the basal body (Fig 2c”, yellow signal). Importantly, co-localization of endogenous NINL and DZANK1 was also observed by immunohistochemistry in rat retina where NINL was demonstrated at the region of the connecting cilium [18]. Using a specific anti-DZANK1 antibody we show that NINL and DZANK1 co-localize in this region (Fig 2d–2f’).

### *Ninl* and *dzank1* genetically interact in photoreceptor outer segment morphogenesis and function

To investigate the biological role of NINL and DZANK1 in the retina, we first addressed the suitability of zebrafish as an animal model. tBLASTn searches of the zebrafish genome, using the amino acid sequence of human NINL and DZANK1, revealed a single ortholog for both genes. Although extensive RT-PCR analyses were performed, we were not able to detect the transcript encoding zebrafish Ninl isoform A in RNA obtained from zebrafish larvae (5 days post fertilization (dpf)). Therefore we conclude that under the given conditions the *ninl* transcript that shows the highest degree of homology with the human NINL^isoB^ encoding transcript is the most prominent. The presence of shorter or alternative transcripts encoding additional zebrafish Ninl isoforms cannot be ruled out, as no 5’- and 3’-RACE experiments were performed. Subsequently, the effect of *ninl* and *dzank1* knockdown during zebrafish embryonic development was investigated, using gene-specific translation-blocking (atgMOs) and splice-blocking morpholinos (spMOs).

**Fig 3:**
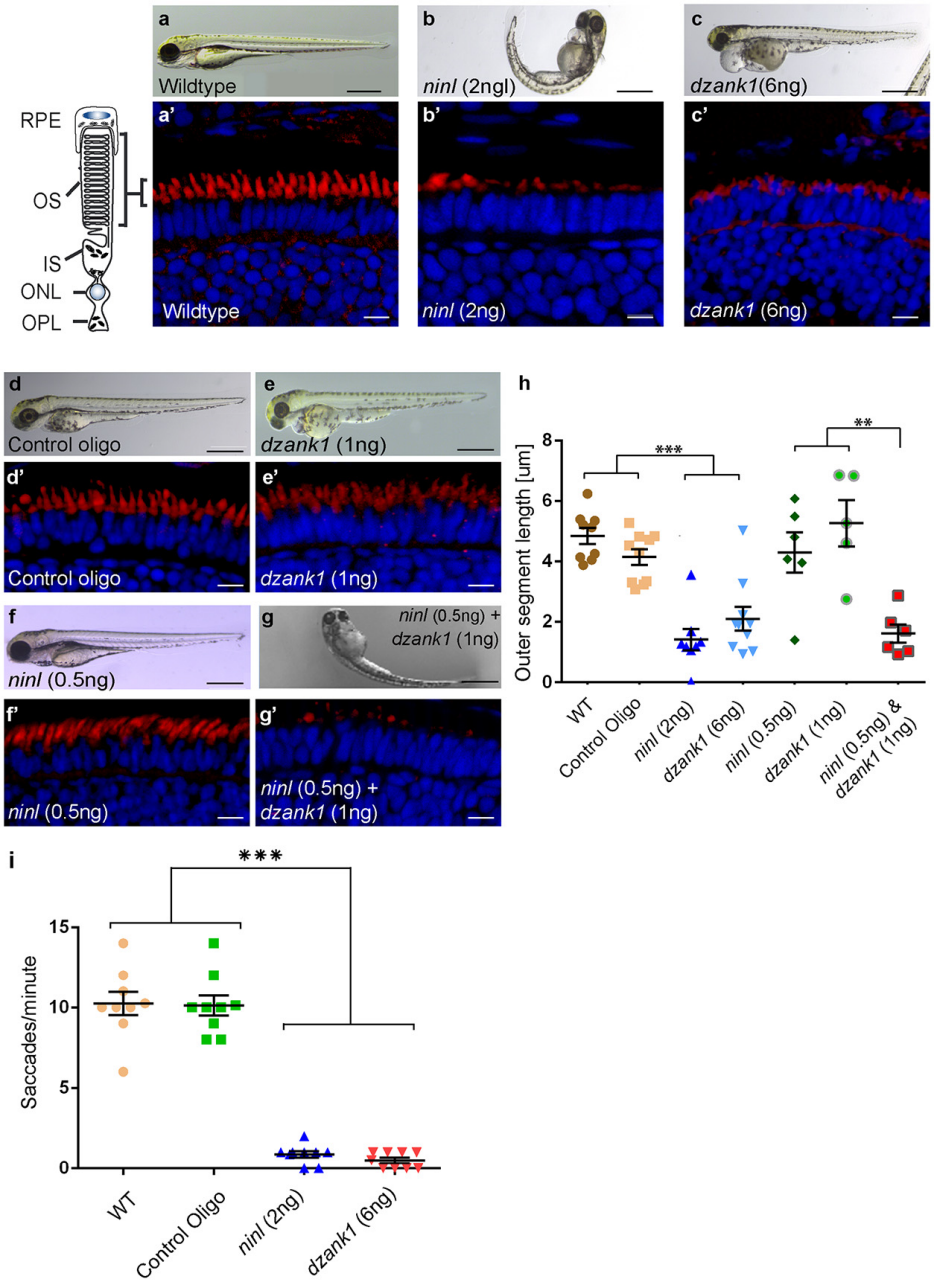
Morphological, functional and epistatic effects of *ninl* and *dzank1* knockdown in zebrafish retina. (a-c) Images of 4 dpf living zebrafish. Un-injected controls (WT) appear morphologically normal (a), while embryos injected with 2 ng of *ninl* atgMO display morphological defects, including ventrally curved body axis and small eyes (b). Embryos injected with 6 ng *dzank1* ex8 spMO resulted in expanded melanophores, small eyes and severe pericardial edema (c) (a’-c’) Retinal histology of 4 dpf zebrafish morphants examined by cryo-sections, where bodipy highlights the OS (red) and nuclei are stained with DAPI (blue) in all panels. Outer segments were shortened and dysmorphic in *ninl* and *dzank1* morphants compared to wildtype larvae. (d-g) *Ninl* interacts genetically with *dzank1*. Injection of sub-effective *dzank1* (1 ng/nl) MO (e’) or *ninl* (0.5 ng/nl) MO (f’) shows normal OS shape and length in morphologically normal appearing larvae, which could not be distinguished from un-injected embryos (WT) or control MO-injected larvae (d’). Combined injection of sub-effective concentrations of *ninl* (0.5 ng/nl) and *dzank1* (1 ng/nl) MO together results in almost complete absence of OS (g’). (h) Quantification of Outer Segment length, shown as a scatter graph where each datapoint represents the mean OS length in one larva, revealed a significantly decreased length of outer segments in *ninl* (2ng/nl), *dzank1* (6ng/nl) and *ninl/dzank1* double morphants as compared to controls. Bars represent the mean value for each treatment group with the Standard error of the mean (SEM) ***P<0.0001, ** *P*<0.001, unpaired Student’s t-test. (i) Analysis of the Opto Kinetic Response (OKR) showing severely decreased responses in larvae injected with 2 ng *ninl* atgMO or 6 ng *dzank1* ex8 spMO (*** p<0.0001, Student’s *t*-test). Scale bars represent 500 μm (a-c and e-h) and 15 μm (a’-c’ and e’-h’). RPE, retinal pigment epithelium; OS, Outer Segment; IS, Inner Segment; ONL, Outer Nuclear Layer; OPL, Outer Plexiform Layer.

Control MO-injected larvae (10 ng/nl; n = 300 from 2 biological replicates) appeared morphologically normal, and could not be distinguished from uninjected larvae (WT) during the studied developmental period of 4 to 5 dpf (Fig 3a and 3d). In contrast, injection of *ninl* atgMO revealed a concentration-dependent spectrum of phenotypes (n>200/group from 2 biological replicates; Bachmann-Gagescu *et al*., companion manuscript). Injection of the optimal concentration of 2 ng/nl *ninl* atgMO resulted in severe morphological defects including ventrally curved body axis, ventriculomegaly, pronephric cysts, expanded melanophores, small eyes and circling swimming behavior at 4 dpf (n = 300 from 2 biological replicates; Fig 3b and Bachmann-Gagescu *et al*., companion manuscript). In addition, we observed significantly shorter photoreceptor outer segments (OS) on retinal cryosections from 4dpf *ninl* morphant larvae stained with boron-dipyrromethene (bodipy) to mark the outer segment membrane disks (mean OS length 1.6+/-0.26 μm in morphants compared to 3.9+/-0.32 μm in wild-type, p<0.0001, unpaired Student’s t-test, n>10 larvae from each group in each of 2 biological replicates) (Bachmann-Gagescu et al, companion manuscript). Co-injection of 150 pg/nl capped MO-resistant mRNA encoding human NINL^isoB^ with 2 ng/nl of *ninl* atgMO rescued the observed body curvature phenotype (curved body shape in 71% of *ninl* atgMO injected larvae (n = 207) versus 36% in *ninl* atgMO + *ninl* mRNA injected larvae (n = 203), data pooled from 2 biological replicates, p<0.0001, two-tailed Fisher’s exact test) and the OS length (mean OS length in rescued larvae 3.8+/-0.25 μm, p<0.0001, Student’s t-test, n = 10 larvae) (Bachmann-Gagescu *et al*, co-submission). In addition, the specificity of the observed phenotypes is further confirmed by the fact that a second morpholino against *ninl* targeting the splice site at the intron14/exon15 junction causing aberrant splicing led to similar phenotypes, including ventriculomegaly, expanded melanophores (4 ng/nl, n = 200) and shortened photoreceptor outer segments as compared to control MO-injected larvae. Finally, using an anti-*ninl* antibody, we observed substantial decrease of Ninl protein on Western blots and on immuno-histochemistry of retinal cryosections for the *ninl* atgMO-injected larvae and milder decrease in both assays for the *ninl* ex15 spMO-injected larvae (Bachmann-Gagescu et al, companion manuscript).

Injection of increasing amounts of a splice-blocking morpholino targeting *dzank1* exon8 resulted in small eyes and severe pericardial edema at 4 dpf (S1a Fig, Fig 3c). In addition, *dzank1* morphants showed impaired ambulatory activity as predicted before [20]. Based on the incidence of the observed phenotypes and non-quantitative RT-PCR analysis on RNA from morphant larvae (harvested at 2 and 4dpf; S1a and S1g Fig.) we determined that the optimal dose of the *dzank1* ex8 spMO was 6 ng/nl. Co-injection of 150 pg/nl capped MO-resistant mRNA encoding human DZANK1 with 6 ng/nl *dzank1* ex8 spMO rescued the observed ambulatory activity phenotype (n = 105, 2 biological replicates; S1b and S1e Fig). This observed ambulatory activity phenotype could be fully recapitulated by a second splice blocking morpholino targeting *dzank1* exon4 (6 ng/nl, n = 162 out of 200 injected larvae from 2 biological replicates; S2 Fig), further indicating the specificity of the used morpholino. Subsequently, we evaluated the retinal morphology of *dzank1* ex8 spMO-treated larvae (4 dpf; n = 40). While retinal lamination was unaffected, significantly shortened photoreceptor outer segments (OS) were observed as compared to controls, as highlighted by boron-dipyrromethene (bodipy) staining of membranes (Fig 3a’–3c’and 3h) (mean OS length 2.1+/-0.39 μm in morphants compared to 4.9+/-0.27 μm in wild-type, p<0.0001, unpaired Student’s *t*-test, n> 10 larvae from each group in each of 2 biological replicates). The observed photoreceptor outer segment defects fully coincided with the ambulatory activity defects observed in *dzank1* ex8 spMO-treated larvae and could be rescued as well (mean OS length in rescued larvae 3.0+/-0.36 μm as compared to 1.9+/-0.25 μm in *dzank1* morphants (*P* = 0.01, unpaired Student’s t-test, n = 13 larvae each group) and 3.5+/-0.25 μm in uninjected larvae (*P* = 0.3 (NS), unpaired Student’s *t*-test, n = 12 larvae)(S1f Fig). These phenotypes could also be recapitulated by a second splice blocking MO targeting *ninl* ex15 or *dzank1* ex4 (Bachmann-Gagescu et al., companion manuscript; S2 Fig). These observations are indicative for an essential role of both Ninl and Dzank1 in OS formation and/or maintenance.

To investigate whether there is a functional relationship between Ninl and Dzank1 in this process, sub-effective doses of *dzank1* ex8 spMO (1 ng/nl) and *ninl* atgMO (0.5 ng/nl) were co-injected. While single injections with these sub-phenotypic amounts of MOs caused no discernible phenotypes (no body curvature defects or abnormal swimming behavior for *ninl* atgMO-injected larvae or no defects in ambulatory activity in *dzank1* ex8 spMO-injected larvae) (n = 200/group, 2 biological replicates; Fig 3e and 3f)), co-injection of both MOs resulted in a severely enhanced phenotype including defects in swimming behavior, small eyes and curved tails (Fig 3d–3g). Furthermore, photoreceptor outer segment length measurements on transverse sections of bodipy-stained retina demonstrated significantly impaired OS formation in double morphants (*ninl* atgMO 0.5 ng/nl + *dzank1* ex8 spMO 1ng/nl; mean OS length 1.6+/-0.3 μm, n = 6), as compared to low dose single *ninl* atgMO-injected (0.5 ng/nl;mean OS length 4.3+/-0.66 μm, n = 6) and *dzank1* ex8 spMO-injected larvae (1ng/nl; mean OS length 5.2+/-0.76 μm, n = 5), and as to control MO-injected (mean OS length 4.2+/-0.26 μm, n = 10) and uninjected (WT) larvae (mean OS length 4.8+/-0.28 μm, n = 9) (Fig 3a’, 3d’, 3e’, 3f’, 3g’ and 3h; *P*<0.001, unpaired Student’s *t*-test for pairwise comparison between double morphants and each low dose single morphant). These retinal defects in double morphants were similar to those quantified in larvae injected with the optimal doses of *dzank1* spMO (6 ng/nl; 2.1+/-0.39 μm, n = 10; Fig 3c’ and 3h) or *ninl* atgMO (2 ng/nl; 1.6+/-0.26 μm, n = 8; Fig 3b’ and 3h) alone. The functional impact of these morphological changes in the morphant retinas was confirmed by measurements of the optokinetic response (OKR) [21, 22], which tests the ability of zebrafish larvae to visually track rotating stripes [23]. At 4 dpf, *ninl* and *dzank1* morphant larvae displayed a grossly impaired OKR response (0–2 saccades per minute), in contrast to the normal OKR response (5–15 saccades per minute) of control morphants and un-injected larvae from the same clutch (mean number of saccades per minute = 10.3 +/- 0.7 in uninjected wild-type, 10.1 +/- 0.6 in control oligo injected, 0.5 +/- 0.2 in dzank1 morphant and 0.9 +/- 0.2 in ninl morphant larvae, n = 9/group; *P*<0.0001 for pairwise comparisons wildtype/control oligo vs each morphant, unpaired Student’s *t*-test) (Fig 3h). Since spontaneous eye movements in the absence of a stimulus were occasionally registered in all MO-injected larvae, the loss of OKR response is not due to a defect in ocular muscular contraction, but to impaired visual function.

### Knockdown of *ninl* and *dzank1* leads to vesicle accumulation in photoreceptor cells

**Fig 4:**
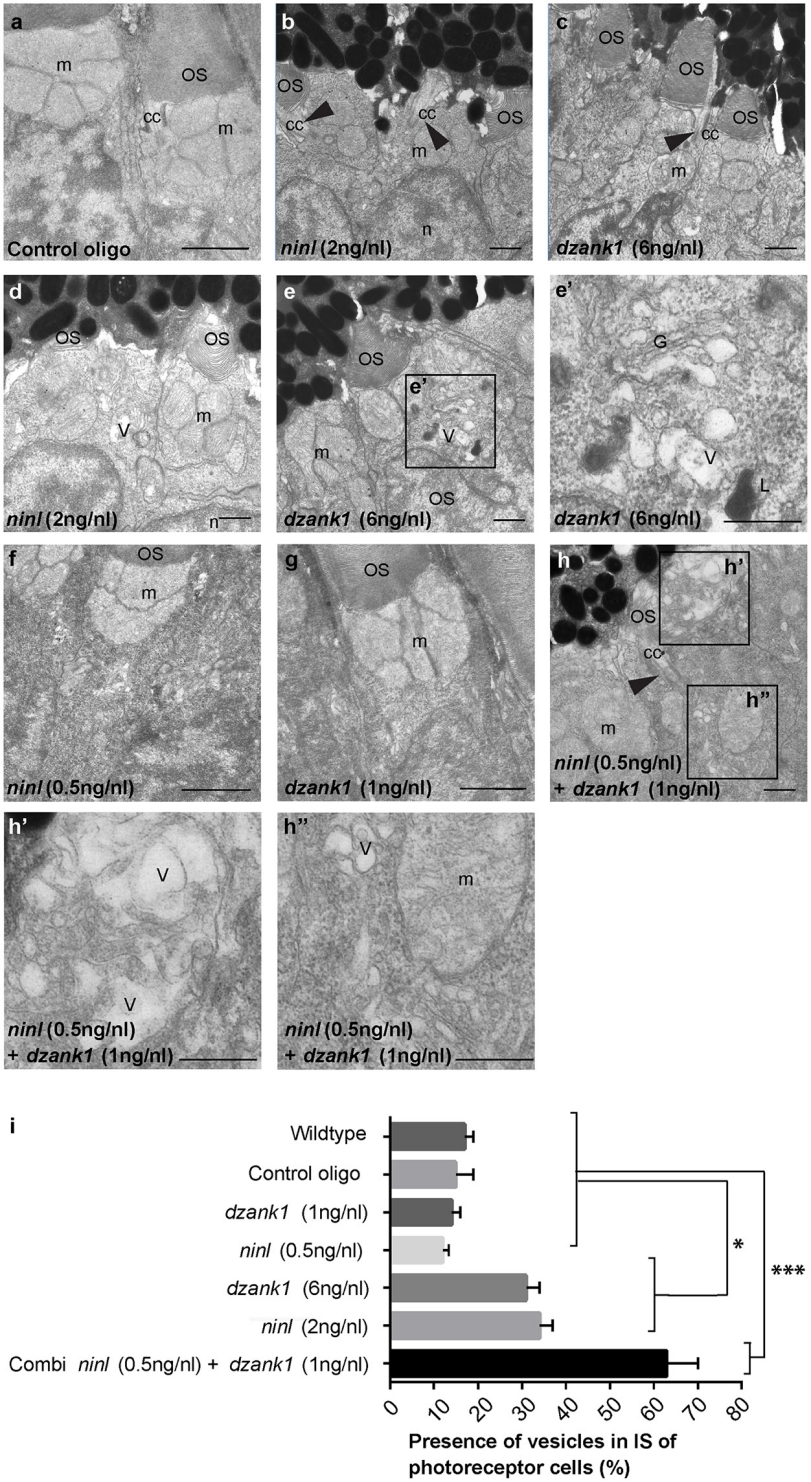
*dzank1* and *ninl* knockdown leads to accumulation of vesicles and vacuoles in zebrafish photoreceptors. Transmission electron microscopy of control (a), *ninl* knockdown larvae (b,d) and *dzank1* knockdown larvae (c,e). *Ninl* and *dzank1* morphants (b-e) demonstrate absent or shortened and dysmorphic outer segments (OS), whereas connecting cilia (CC) are still intact (arrowheads in b-c,h), and accumulation of vesicular structures (v), highlighted in the boxed area in e (e’). Sub-effective concentration of *ninl* (f) and *dzank1* (g) MO-injection leads to a normal phenotype compared to control MO-injected larvae (a) and uninjected controls. Injection of combined sub-effective concentrations of *dzank1* and *ninl* (h) MO leads to increased vesicle accumulation in OS (h’) and inner segment (h”). (i) Analysis of the presence of vesicles in inner segments of photoreceptor of cells (%). On the Y—axis the different classes are indicated (minimum of 6 eyes per group). (* *P*<0.01 and *** *P*<0.001 Student’s *t*-test). Larvae in all panels are 4 dpf. Scale bars represent 0.5 μm. OS: outer segment, CC: connecting cilium, m: mitochondria, n: nucleus, v: vesicular structures, G: Golgi system, L: lysosome.

To obtain a more detailed insight into the role of Ninl and Dzank1 in OS morphogenesis, the retinal ultra-structure of *ninl*, *dzank1* morphants and control larvae was studied by transmission electron microscopy (TEM) (for numbers see S5 Table). In control MO-injected embryos (4 dpf), the photoreceptor inner segments (IS) displayed compact ellipsoid regions with clustered mitochondria, a narrow myoid with a Golgi apparatus, while OS presented well-organized, nicely stacked disc structures (Fig 4a), as described for wild-type uninjected larvae [17, 24]. In contrast, in both *ninl* and *dzank1* morphants, IS malformations were found including swollen Golgi complexes with enlarged and distended cisternae, accumulation of vesicle-like structures throughout the IS, large vacuoles and dispersed mitochondria. Occasionally, lysosomal structures were observed (Fig 4e’). OS were either absent, or disrupted. Ultra-structural characteristics of deviant OS were hampered elongation, accumulation of vesicles, polarization defects and deformed discs (Fig 4d and 4e’). Statistical analyses of these defects were based on quantification of the proportion of photoreceptor cells presenting with vesiculated inner segments in the different experimental groups (Fig 4i). No significant difference could be demonstrated between vesiculation in IS of larvae injected with sub-phenotypic doses of *dzank1* (1 ng/nl) or *ninl* (0.5 ng/nl) MO (Fig 4f and 4g) as compared to control MO-injected (10 ng/nl) or wildtype (WT) zebrafish larvae. The percentage of IS in photoreceptors with vesicular structures and/or swollen Golgi complexes in *dzank1* (6 ng/nl MO), *ninl* (2 ng/nl MO) as well as combined (*dzank1* 1 ng/nl and *ninl* 0.5 ng/nl) MO-injected zebrafish groups was significantly increased compared to WT and control MO-injected larvae (*P*<0.01, Student’s *t*-test). The most significant vesicular increase was observed in the combined MO-injected (*dzank1* and *ninl*) group (Fig 4h and 4h”) (*P*<0.001, Student’s *t*-test).

### NINL and DZANK1 associate specifically with complementary subunits of the cytoplasmic dynein 1 motor complex

**Fig 5:**
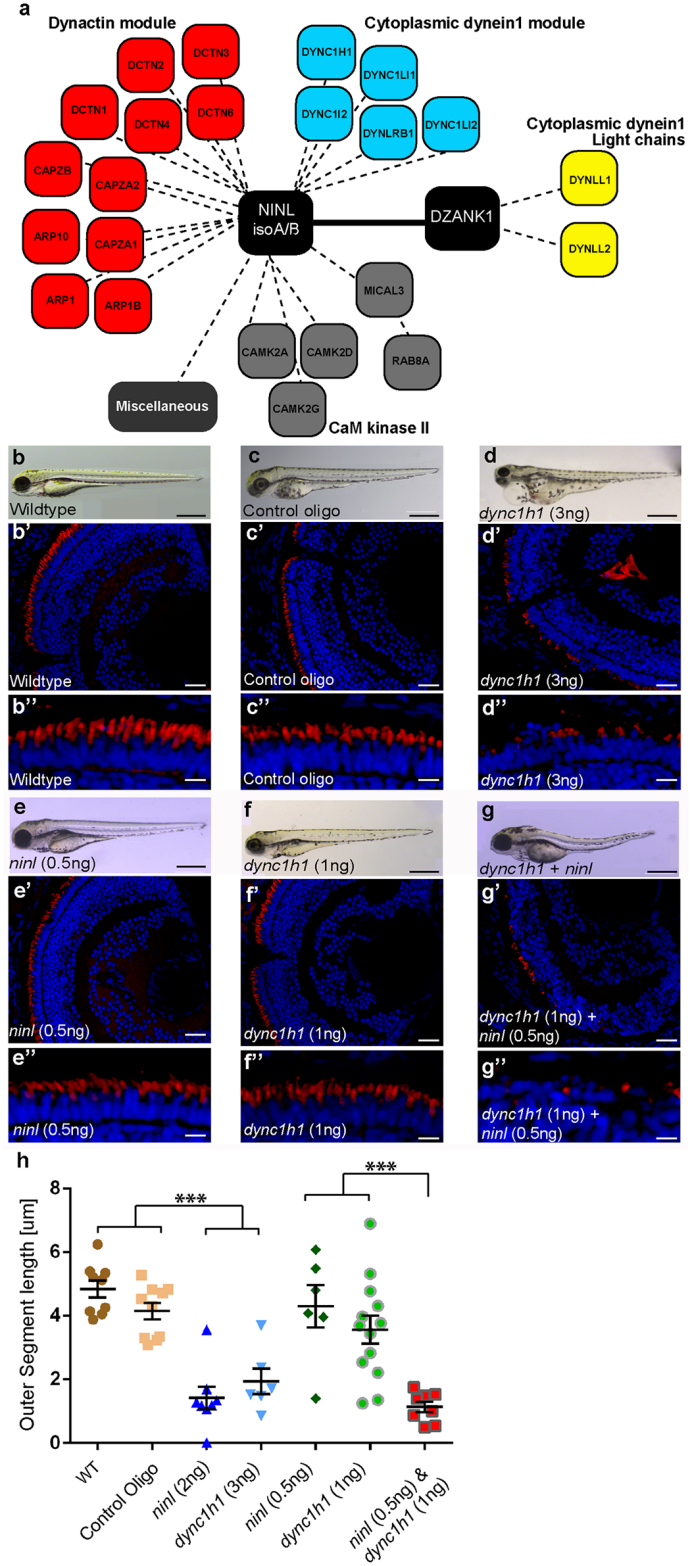
NINL and DZANK1 associate specifically with complementary subunits of the cytoplasmic dynein 1 motor complex. (a) Strep-SILAC and TAP experiments show that DZANK1 interacts specifically with DYNLL1 and DYNLL2 (yellow). NINL associates with components of the dynactin complex (red) and with most components of the dynein 1 motor complex (blue), except for DYNLL1 and DYNLL2. The solid line between NINL and DZANK1 symbolizes a direct interaction, whereas the dashed lines stand for interactions determined by immune and affinity purifications. (b-g”) Genetic interaction between *ninl* and *dync1h1*. MO-injection with a high concentration of *dync1h1* (3 ng/nl) shows larvae with pericardial edema, small eyes and short OS (d-d”). MO-injection of sub-effective concentrations of *ninl* (0.5 ng/nl) (e-e”) or *dync1h1* (1 ng/nl) (f-f”) generates larvae with a normal phenotype and normal OS length, compared to wildtype larvae (b-b”) and control MO-injected larvae (c-c”). The combination of sub-effective MO concentrations of *ninl* (0.5 ng/nl) and *dync1h1* (1 ng/nl) results in short larvae and virtual absence of outer segments (g-g”). All larvae are 4 dpf. Panels b’-d” and e’-g” are retinal cryo-sections stained with bodipy (red) to highlight the outer segments and DAPI to stain the nuclei. (h) Quantification of photoreceptor outer segment length revealed a significantly decreased length of outer segments in *dync1h1* (3ng/nl) and *dync1h1/ninl* double morphants as compared to controls (*** *P*<0.0001; two-tailed, unpaired Student’s *t*-test). Scale bars represent 500 μm (b-g), 50 μm (b’-g’) and 15 μm (b”-g”).

Mass spectrometry (MS)-based quantitative proteomics was employed to gain further insights into the molecular basis of the defects observed in *ninl* and *dzank1* morphants. N-terminally fused Strep/FLAG-tagged NINL^isoA/B^ and DZANK1 together with their associated, native protein complexes were tandem-affinity-purified from HEK293T cells and the complex components identified by liquid chromatography coupled to tandem mass spectrometry (LC-MS/MS). Besides six actin-binding proteins (ARP1, ARP1B, ARP10, CAPZA1, CAPZA2 and CAPZB) and three subunits of Ca^2+^/calmodulin-dependent protein kinase II (CaMKII) (CAMK2A, CAMK2D, and CAMK2G), the NINL-associated interactome contained multiple subunits of the cytoplasmic dynein 1-dynactin motor complex (DYNC1H1, DYNC1LI1, DYNC1LI2, DYNCI2, DYNLRB1, DCTN1-4, and DCTN6), which is involved in minus end–directed, microtubule-associated transport (Fig 5a, S2 Table). These results were confirmed by the GST pull-down from bovine retinal extracts in which DZANK1, several subunits of the cytoplasmic dynein 1-dynactin motor complex (DYNC1H1, DYNC1LI1, DCTN1, DCTN2 and DCTN4) and five dynactin-associated actin-binding proteins (ARP1B, ARP10, CAPZA1, CAPZA2 and CAPZB) were found to associate with GST-fused NINL^isoB^_aa538-825 (S1 Table). We thus confirmed the previously described association of NINL with several subunits of the cytoplasmic dynein 1-dynactin motor complex [19]. Intriguingly, the DZANK1-associated protein complex exclusively contained the two cytoplasmic dynein 1 light chains, DYNLL1 and DYNLL2, which were absent from the NINL interactome. We confirmed the identified interaction between DZANK1 with DYNLL1 and DYNLL2 by reciprocal co-IP experiments in HEK293T cells. In addition, co-expression of mRFP-tagged DYNLL1 or DYNLL2 with eCFP-tagged DZANK1 in ciliated hTERT-RPE1 cells resulted in recruitment of the latter to the basal body and accessory centriole (S3 and S4 Figs). These results suggest that the cytoplasmic dynein 1 motor complex is composed of at least two sub-complexes: one DZANK1-associated sub-complex containing DYNLL1 and DYNLL2, and another NINL-associated sub-complex containing at least DYNC1H1, DYNC1LI1, DYNC1LI2, DYNCI2 and DYNLRB1. In order to better understand the orchestration and dynamics of the NINL-associated motor complex, we performed an elution profile analysis of SDS-induced sub-complexes by quantitative mass spectrometry (EPASIS) [25]. SF-TAP-tagged NINL^isoA/isoB^ was over-expressed in HEK293T cells and affinity-purified using the FLAG moiety of the fusion tag. The native protein complex was sequentially treated with increasing SDS-concentrations to destabilize the interactions and thereby induce the elution of sub-complexes. Besides the dynactin submodule, which showed the most stable association with the NINL complex (S5c Fig; complete elution at ≥ 0.005% SDS) a second sub-module consisting of proteins from the cytoplasmic dynein 1 motor complex [26] was identified (S5c Fig c; elution between 0.001 and 0.01% SDS). This dynein sub-module may dissociate in two additional sub-modules but this finding lacked statistical significance (S5–S8 Figs, S3 and S4 Tables). In addition, CLIP1 (= CLIP-170), PAFAH1B1 (= LIS1), ACAD11 and MRPS27 co-eluted with the dynein module, making them potentially novel candidate components of the dynein 1 motor complex.

### Zebrafish Ninl and Dzank1 are required for intracellular dynein-based transport

*Dynein 1 heavy chain 1* (*dync1h1*), encoding a subunit of the cytoplasmic dynein 1 motor complex which mediates minus-end-directed post-Golgi vesicle trafficking towards the basal body, is mutated in the zebrafish *cannonball* mutant [17]. Similar to our findings in *ninl* and *dzank1* morphants, *cannonball* mutants form short rudimentary photoreceptor OS, show organelle positioning defects and display a severe accumulation of vesicles in the photoreceptor IS and OS and this phenotype could be recapitulated by a translation blocking morpholino against *dync1h1* [17]. Sub-effective concentrations of this previously published *dync1h1* MO (1 ng/nl) [17] in combination with the *ninl* atgMO (0.5 ng/nl) were co-injected and compared to single injected morphants, control MO-injected larvae and uninjected controls (n = 300/group from 2 biological replicates). At 4 dpf, quantification of OS lengths in each group was performed on bodipy-stained cryosections and revealed significantly shortened OS in larvae in the *dync1h1* (3 ng/nl; mean OS length 1.95+/-0.4 μm, n = 5; Fig 5d’, 5d” and 5h) and *dync1h1/ninl* morphant groups (1ng/nl + 0.5 ng/nl; mean OS length 1.413+/-0.2 μm, n = 13; Fig 5g’, 5g” and 5h) as compared to uninjected controls (mean OS length 4.8+/-0.27 μm, n = 9; Fig 5b’, 5b” and 5h), control MO-injected (10 ng/nl; mean OS length 4.2+/-0.26 μm, n = 10; Fig 5c’, 5c” and 5h) and to both low-dose single *dync1h1* MO-injected (1 ng/nl; mean OS length 3.6+/-0.44 μm, n = 13; Fig 5f’, 5f” and 5h) and *ninl* atgMO-injected (0.5 ng/nl; mean OS length 4.3+/-0.66 μm, n = 6; Fig 5e’, 5e” and 5h) groups (*P*<0.001; two tailed, unpaired Student’s *t*-test on pairwise comparisons between double morphants and each of the single low-dose morphants), demonstrating a genetic interaction between *ninl* and *dync1h1* and supporting a role for Ninl in cytoplasmic dynein 1-driven intracellular vesicle transport. In addition, localization of rhodopsin, which should be restricted to the OS, was aberrantly observed in the photoreceptor cell body in both *ninl* and *dzank1* morphants (S9 Fig and Bachmann-Gagescu *et al*. co-submission.).

**Fig 6:**
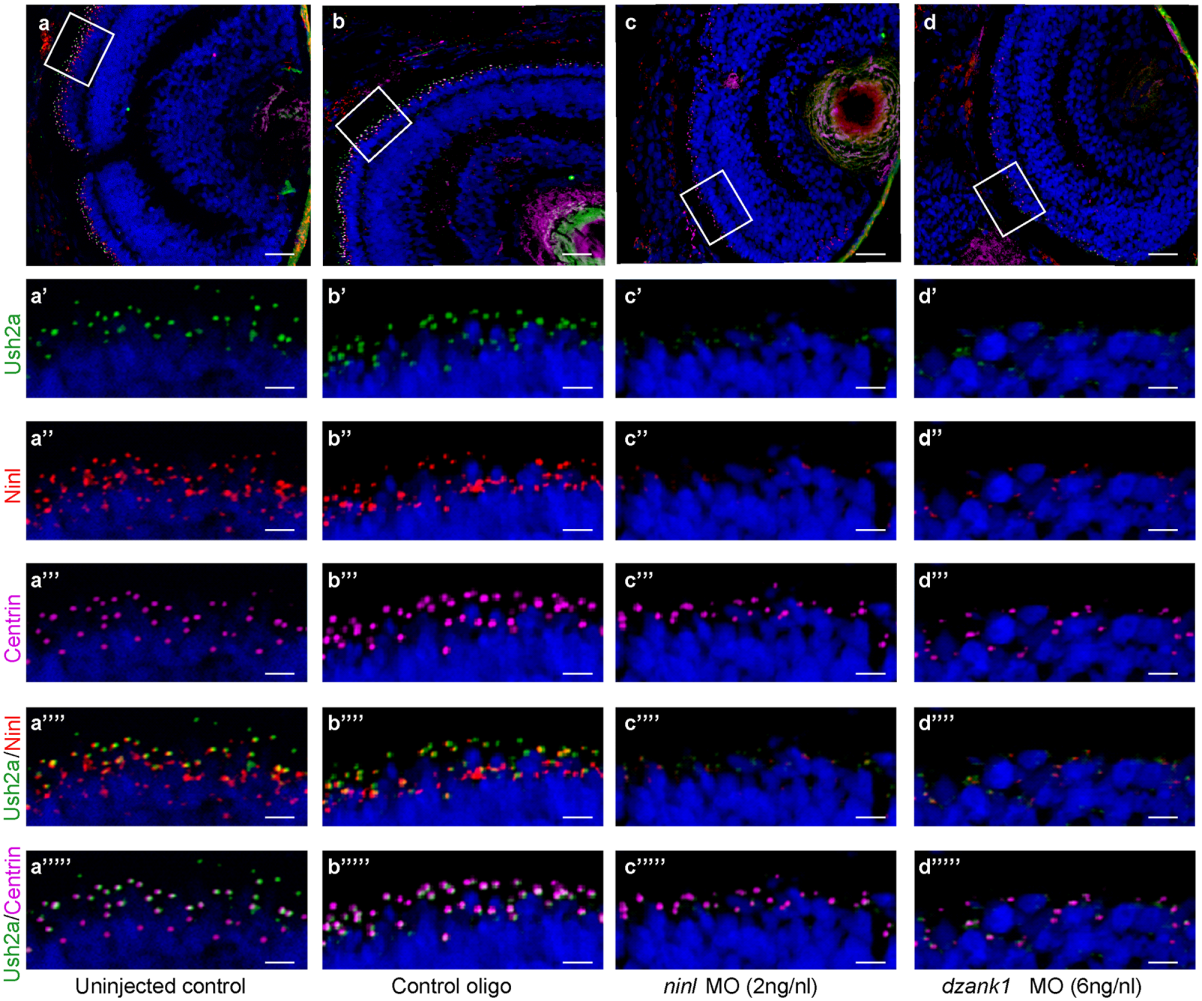
Impaired Ush2a transport in *dzank1* and *ninl* morphants. (a-d) Overview of Ninl and Ush2a localization in cryo-sections of 4 dpf zebrafish retina using anti-Ninl (red signal), anti-Ush2a (green signal) and the basal body marker anti-Centrin (cyanid signal). In all images the nuclei are counterstained with DAPI (blue signal). (a-a”“‘) Un-injected wildtype, (b-b”“‘) 10 ng/nl control MO-injected, (c-c”“‘) 2 ng/nl *ninl* MO-injected and (d-d”“‘) 6 ng/nl *dzank1* MO-injected zebrafish retinas. (a’-d’) Anti-Ush2a staining (green signal) is strongly reduced in *dzank1* and *ninl* morphants (c’-d’), while Ush2a is clearly present in wildtype and control MO-injected larvae. (a”-d”). Specific Ninl-immunofluorescence (red signal) was largely abolished in *ninl* morphants and reduced in *dzank1* morphants. (a”‘-d”‘) Centrin (cyanide signal) was observed in wildtype, un-injected control and in both *ninl* and *dzank1* morphants. (a”“-d”“) Co-localization of Ush2a and Ninl (yellow signal) was observed in wildtype and control MO-injected larvae. (a”“‘-d”“‘) Co-localization of Ush2a and Centrin (yellow signal) was seen in all images (WT, Control Oligo, *ninl* and *dzank1* morphants), despite strong reduction of Ush2a immunofluorescence in *ninl* and *dzank1* morphants. Scale bars represent 15 μm, except for (a-d) in which the scale bars represent 50 μm.

The observed mislocalization of rhodopsin in these morphants could be explained by defects in intracellular transport. However it could also be the result of defects in OS development and a consequence of the absence of this structure. Although the OS of *ninl* morphants are severely affected, the connecting cilium of photoreceptor cells in *ninl*, *dzank1* and *ninl-dzank1* double morphants was found to be intact (arrow heads in Fig 4b, 4c and 4h), providing the opportunity to test the localization of proteins to this ciliary compartment regardless of the presence of an outer segment. USH2A, a previously published interaction partner of NINL, is a transmembrane protein that is synthesized in the IS and subsequently transported in TGN-derived vesicles towards the base of the connecting cilium. USH2A was previously shown to localize in the peri-ciliary region, which should be independent of the presence of the OS [27]. Therefore, a defect in USH2A localization would likely be due to impaired post-Golgi dynein-based trafficking. Using a zebrafish-specific anti-Ush2a antibody, we observed a strongly reduced immunofluorescent signal in *ninl and dzank1* morphants (4 dpf), as compared to control MO-injected larvae (4 dpf) (Fig 6a’–6d’). The localization of centrin, which was used as a marker for the connecting cilium, was unaffected in both controls and morphants (Fig 6a”’–6d”’). Together, these data indicate a defect in the transport of vesicle-bound transmembrane proteins in *ninl* and *dzank1* morphants.

**Fig 7:**
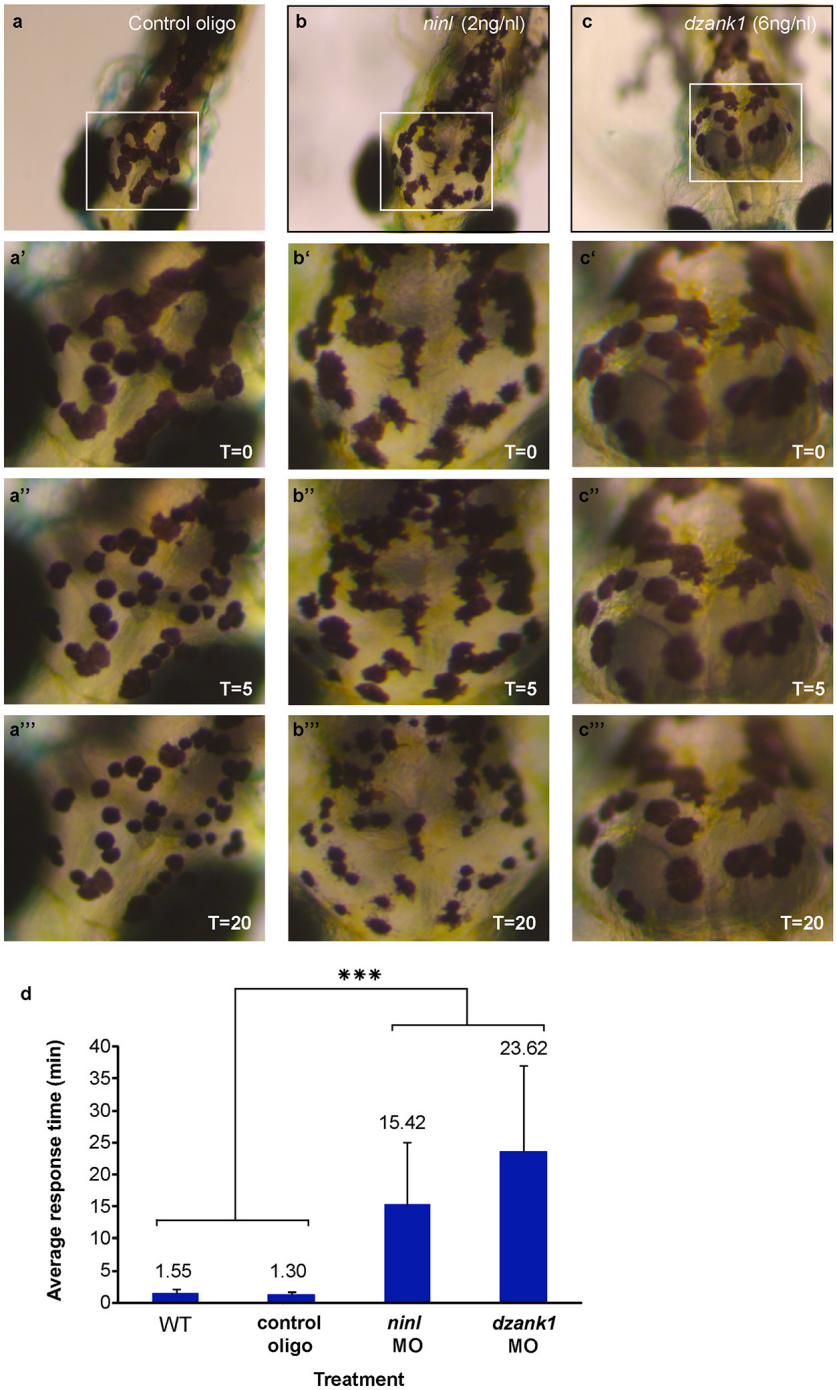
*ninl* and *dzank1* knockdown results in epinephrine-induced melanosome retraction delay. Control MO-injected larvae (10 ng/nl; n = 20) at 5 dpf (a-a”‘), *ninl* morphant (2 ng/nl; n = 20) (b-b”‘) and *dzank1* morphant (6 ng/nl; n = 20) (c-c”‘). White Box denotes the area at higher magnification (40x) (a’-c”). (a-c’) Melanosome pattern of the different larvae before treatment, (a”-c”), 5 min after epinephrine addition and (a”‘-c”‘) 20 min after epinephrine addition, t represents time in minutes. (d) Graphical representation with error bars (standard deviation) demonstrating a significant delay of epinephrine-induced melanosome retrograde trafficking compared with wild-type and control MO-injected (10 ng/nl) larvae. Treatment (control, *ninl*- or *dzank1*-morphants) is noted on the *x*-axis and average response time in minutes is noted on the Y-axis. ***: P<0.001 (two-tailed, unpaired Student’s *t*-test).

To determine whether Ninl and/or Dzank1 are required for other dynein-based intracellular transport processes, we monitored dynein 1-mediated melanosome transport in zebrafish skin. Zebrafish alter their skin pigmentation by trafficking melanosomes within melanophores. The melanosome, a lysosome-related organelle, can be shuttled bi-directionally between the cell periphery and the peri-nuclear region by two microtubule-based molecular motors, kinesin ll (anterograde) and dynein 1 (retrograde). Pigment aggregation (retrograde transport) can be stimulated within minutes upon treatment with epinephrine [28]. In the melanosome transport assay, 5 dpf larvae are dark-adapted to display maximum melanophore dispersion (Fig 7a). After addition of epinephrine, the melanosomes rapidly contract and can be visually evaluated for reduction in pigment dispersion (Fig 7a’–7a”). The endpoint is apparent when pigmentation pattern reflects peri-nuclear accumulation of melanosomes (Fig 7a’). A rapid melanosome contraction was seen in control MO-injected and un-injected larvae (Fig 7a–a” and 7d) (ΔT = 1.55 min and ΔT = 1.30 min respectively; n = 20/group), whereas *ninl* and *dzank1* morphants demonstrated significantly delayed dynein 1-mediated melanosome retraction (ΔT = 15.42 min and ΔT = 23.62, n = 20; *P*<0.001; Student’s *t*-test (two tailed, unpaired), Fig 7b–7c”’ and 7d), which is indicative for impaired dynein 1-mediated retrograde transport. Taken together, our findings suggest that the Ninl-Dzank1-cytoplasmic Dynein 1 complex is required for the intracellular transport of organelles and vesicles, and is essential for the photoreceptor’s OS formation, maintenance and function.

## Discussion

In this study, a central role for NINL and its novel interaction partner DZANK1 is identified in vesicle transport in photoreceptor cells. We demonstrate that NINL and DZANK1 associate with complementary subunits of the cytoplasmic dynein 1 motor complex and that this complex consists of at least two submodules. In photoreceptor cells, this motor complex has been implicated in post-Golgi vesicle trafficking and organelle positioning [14, 17]. In line with this function, *in vivo* studies in the zebrafish demonstrate defects in post-Golgi trafficking as revealed by delayed cytoplasmic dynein 1-regulated melanosome transport, defective photoreceptor outer segment formation, abnormal vesicle accumulation within the photoreceptor inner segments and mislocalization of rhodopsin and Ush2a in both *ninl* and *dzank1* morphants.

Cytoplasmic dynein 1 is fundamentally important for embryonic development. Dynein 1 heavy chains are essential for the formation of the motor complex and their absence leads to very early embryonic lethality in mice before E8.5 [29]. In contrast, larvae of the zebrafish *cannonball* (*cnb*) mutant lacking Dync1h1 undergo relatively normal early development and remain indistinguishable from wild-type siblings until approximately 3.5 dpf. This remarkable difference between mouse and zebrafish mutants may be explained by the presence of wild-type maternally-derived (yolk-associated) mRNA in zebrafish embryos [30]. *Cnb* larvae eventually show a reduced eye size, present with small rudimentary photoreceptor outer segments and expanded skin melanophores, show severe organelle positioning defects and die between 6 and 8 dpf [17]. The phenotype of zebrafish depleted for Ninl is remarkably similar to that of the *cnb* mutant, with small eyes, mispositioned organelles, retinal dystrophy, and expanded melanophores. These overlapping phenotypic characteristics in combination with the observed genetic interaction in the retina between *ninl* and *dync1h1* suggest that the *ninl* morphant phenotype is caused by dysfunctional cytoplasmic dynein 1-mediated transport.

Mice lacking Dync1li1, a light-intermediate chain subunit of dynein 1, which is structurally less important than heavy chains, survive into adulthood [31]. Their photoreceptors do, however, lack outer segments due to blocked transport of TGN-derived vesicles towards the basal body. In contrast to the *ninl* morphants, *dzank1* morphants show a much milder phenotype and do not present curved bodies or major early developmental defects. DZANK1 associates specifically with two structurally less important dynein 1 light chains, which might explain the less severe phenotype of *dzank1* morphant zebrafish larvae. Nonetheless, photoreceptor cells of both *ninl* and *dzank1* morphant zebrafish larvae display severely shortened outer segments, disruption of mitochondria organization and accumulation of vesicles within the inner segments. Given that photoreceptors have extremely high transport requirements due to the significant daily renewal of their outer segments, they are predicted to be more sensitive to defects in intracellular transport, which could explain why loss of *dzank1* results predominantly in a photoreceptor phenotype.

The physical position and role of NINL and DZANK1 in the cytoplasmic dynein 1 motor complex remains unknown. Up to now, tandem affinity purification assays from HEK293T cells using different subunits of this motor complex as bait never identified peptides of NINL or DZANK1 (S2 Table). The most likely explanation for this is that NINL and DZANK1 are not expressed in these cells or are expressed only at very low levels. To get the first insights into the dynamics and orchestration of the NINL-associated dynein 1 motor complex, we performed an elution profile analysis of SDS-induced sub-complexes by quantitative mass spectrometry (EPASIS). Despite the fact that the dynein module shows a tendency of being built up of two distinct sub-modules, the statistical significance is lacking. CLIP1 (= CLIP-170), PAFAH1B1 (= LIS1), ACAD11 and MRPS27 co-eluted with the dynein module, making them potentially novel candidate components of the dynein 1 motor complex. LIS1 was previously found to interact with cytoplasmic dynein 1-dynactin [32] and CLIP1 [33] in order to keep dynein in a persistent microtubule-bound state [34]. The role of ACAD11 and MRPS27 in dynein 1 function or dynamics needs to be determined. The dynactin complex was identified as a distinct sub-complex which was most strongly associated with NINL in the EPASIS essay. The lack of clear OS defects in zebrafish *mok/dctn1a* and *dctn1b* mutants [17, 35, 36] however, implies that dynein 1 functions independently of dynactin in outer segment morphogenesis. Indeed it was reported that the binding of dynactin1 to dynein 1 is non-essential for the ability of dynein 1 to bind stably to rhodopsin transport vesicles *in vitro* [14]. Therefore it can be concluded that the ocular phenotypes observed in the *ninl* morphant are most likely caused by a non-functional dynein 1 complex rather than dysfunction of the dynactin complex.

We previously described the association of NINL^isoB^ with USH2A [18], a protein known to be essential for photoreceptor homeostasis in mice [27, 37]. The retinal defects observed in *ninl* morphants are more severe than those observed in the *Ush2a* knockout mouse model, which displays intact outer segments and late-onset mild photoreceptor degeneration [27]. This comparison suggests a transport function for NINL^isoB^ upstream of USH2A towards the apical inner segment. The absence of Ush2a at the photoreceptor periciliary region of *ninl* morphants is in line with this hypothesis.

DZANK1 protein sequence analysis predicted the presence of two ZNF_RBZ domains and their interaction with RanGDP rather than their involvement in transcription. Ran is an abundant Ras-like GTPase, which plays a role in multiple cellular processes, including modulation of nucleo-cytoplasmic transport of macromolecules larger than ~40 kDa across the nuclear envelope [38]. Further, it has been proposed that a similar complex, consisting of Ran, Ran-binding proteins and importins/exportins plays a role in regulating import of cargo at the base of the cilium [39] and that RanBP2 is involved in processing or transport of opsin [40]. Since the ZNF domains of DZANK1 are highly homologous to the functional domains of RanBP2, DZANK1 might be involved in transport of opsin as well. The observed mislocalization of rhodopsin in photoreceptor cells of *dzank1* morphant zebrafish larvae is in line with this. Moreover, a role for DZANK1 in opsin transport and subsequent ciliary entry creates an attractive functional connection between DZANK1 and the Usher protein network, members of which have been suggested to act in opsin vesicle docking at the periciliary region and subsequent transport in the connecting cilium and calyceal processes [41, 42].

In summary, our study provides a deeper insight into the tissue-specific dynamics of the cytoplasmic dynein 1 motor complex, and supports an essential role for this complex in close connection to NINL and DZANK1 in post-Golgi vesicle transport of selective cargo in zebrafish photoreceptor cells.

## Methods

### Ethics statement

Animal experiments were conducted in accordance with the Dutch guidelines for the care and use of laboratory animals, with the approval of the Animal Experimentation Committee (Dier Experimenten Commissie [DEC]) of the Royal Netherlands Academy of Arts and Sciences (Koninklijke Nederlandse Akademie van Wetenschappen [KNAW] (Protocol # RU-DEC 2012–301).

### Yeast two-hybrid interaction assay

To identify the interacting regions between DZANK1 and NINL^isoA/B^, a GAL4-based Y2H screen (HybriZAP, Stratagene, La Jolla, CA, USA) was used as previously described [43, 44]. Accession IDs: NINL^isoA^ (Q9Y2I6), NINL^isoB^ (XP_005260736), DZANK1 (Q9NVP4), DYNLL1 (NP_001032584), DYNLL2 (Q96FJ2).

### Localization studies in cells

The cellular (co)-localization of DZANK1, NINL^isoB^, DYNLL1 and DYNLL2 was determined by co-transfecting hTERT-RPE1 cells on glass slides, with pcDNA3-mRFP and pcDNA3-eCFP. DZANK1 FL, NINL^isoB^, DYNLL1 and DYNLL2 were transfected using Effectene Transfection Reagent (Qiagen, Netherlands) according to manufacturer’s instructions. After 48 hours transfection, cells were washed with PBS, fixed with 4% paraformaldehyde (PFA) and mounted with Vectashield containing DAPI (Vector Laboratories, Inc., UK). Images were taken with an Axioplan2 Imaging fluorescence microscope (Zeiss) and processed using Adobe Photoshop version 8.0 (Adobe Systems, USA).

### GST pull-down

The GST-fusion proteins were produced by transforming *Escherichia coli* BL21-DE3 with plasmid pDEST15-NINL (538aa to 825aa), as previously described [43]. Strep/FLAG-tagged DZANK1 or Strep/FLAG-tagged NINL^isoB^ were produced by transfecting HEK293T cells with plasmids encoding N-SF-TAP-hsDZANK1 or NINL^isoB^, respectively, using the transfection reagent (PEI; Polyethylenimine), according to the manufacturer's instructions. The GST pull-down assay was performed as described previously [43]. For GST pull-down experiments from retinal extracts, retinas were dissected from fresh bovine eyes obtained from the local slaughter house. Retinas were homogenated by sonication on ice for two times 30 s in extraction buffer [10 mM HEPES (pH 7.9), 10 mm NaCl, 3 mm MgCl_2_, freshly added 1 mm DTT, 1 mm Na_3_VO_4_], supplemented with complete protease inhibitor cocktail (Roche Diagnostic). Retinal extracts were incubated overnight at 4°C with the GST fusion proteins immobilized on glutathione sepharose 4B beads. GST fusion proteins were eluted from the glutathion sepharose 4B beads with 100 mM reduced Glutathione (GSH) in 50mM TRIS-HCl (pH 8.0) overnight. Proteins were subsequently precipitated and analyzed by mass spectrometry analysis as described below.

### Co-immunoprecipitation in HEK293T cells

HA-tagged NINL and DYNLL1 were expressed by using the mammalian expression vector pcDNA3-HA/DEST, the 3xHA-tagged DYNLL2 by using p3xHA_CMV/DEST, Strep/FLAG-tagged DZANK1 by using pSF-NTAP/DEST and LRRK2 by using p3xFLAG/DEST from the Gateway cloning system (Invitrogen, USA). HEK293T cells were co-transfected, using Effectene Transfection Reagent (Qiagen, USA) according to the manufacturer’s instructions. Twenty-four hours after transfection, the cells were washed with PBS and subsequently lysed in IP lysis buffer (50 mM Tris-HCL pH 7.5, 150 mM NaCl, 1% Triton-X-100 supplemented with complete protease inhibitor cocktail (Roche, Germany)) on ice. HA-tagged molecules were immune-precipitated from cleared lysates at 4°C overnight. Protein-antibody complexes were coupled to ProtA/G beads (Santa Cruz) for 2 hours at 4°C. After incubations, the beads were pelleted and washed three times with lysis buffer. Beads were boiled and proteins were resolved on SDS-PAGE. For western blotting, proteins were electrophoretically transferred onto nitrocellulose membranes, blocked with 5% non-fat dry milk (Biorad) in PBS-T (PBS supplemented with 0.1% Tween) and analyzed with the appropriate primary and secondary antibodies in 0.5% non-fat dry milk in PBS-T. After 4 washes in lysis buffer, the protein complexes were analyzed on immunoblots using the Odyssey Infrared Imaging System (LI-COR, USA). As secondary antibodies, IRDye800 goat-anti-mouse IgG and Alexa Fluor 680 goat-anti-rabbit IgG were used.

### Antibodies

The monoclonal antibodies directed against Centrin (1:100), Millipore, lot nr: 04–162 and the polyclonal antibodies directed against the cytoplasmic region of USH2A^isoB^ (1:100), Novus Biological, lot nr: T00620A02 have been described previously [43, 45]. For the rhodopsin staining anti-rhodopsin, clone 4D2 Millipore lot nr: 2038649 (1:1000) was used.

For Western blot and immunohistochemical analyses, antibodies directed against human NINL (aa406-455) were purchased from LSBio, cat. No. LS-C201509 (1:100). Antibodies against the C-terminal region of zebrafish Ninl, which were raised in guinea pigs against a GST-fusion protein, encoding a peptide consisting of 403aa to 591aa (Genbank NP_001268727) were used for immunohistochemical analyses. The cDNA, encoding this peptide was amplified by using the forward and reverse primers 5'-GACCAAGCCTGTCAAGAGCG-3' and 5'-GCCCTGAGACTTCAACAAC-3', respectively. The secondary antibodies were goat anti-guinea pig Alexa Fluor 488 and Alexa Fluor 568, goat anti-rabbit Alexa Fluor 488, goat anti-mouse Alexa Fluor 488, Alexa Fluor 568 and Alexa Fluor 647; (all used at a dilution of 1:500, all from Molecular Probes-Invitrogen Carlsbad, CA, USA). The latter were diluted together with DAPI in block buffer (2% BSA and 10% Normal Goat serum in PBS).

### Zebrafish

Experimental procedures were conducted in accordance with international and institutional guidelines. Wild type adult Tupfel Long fin (TLF) zebrafish were used. The zebrafish eggs were obtained from natural spawning of wild-type breeding fish. Larvae were maintained and raised by standard methods [46]. Translation-blocking *ninl* (5’-CATCCTCGTCCATCCCACCACATAC-3’), exon15 splice-blocking *ninl* (5’- CCCAACACTAAAGAGATACACCAAT-3’), exon4 splice-blocking *dzank1* (5’- CGGCCATCACTGCATCACATTACAA-3’) exon8 splice-blocking *dzank1* (5’- AGGACATCTTTAGAATGATAGACGT-3’) and translation blocking *dync1h1* (5’- CGCCGCTGTCAGACATTTCCTACAC-3’) morpholinos (MOs) were designed by Gene Tools Inc. (USA) and diluted to the appropriate concentration in deionized, sterile water, supplemented with 0.5% phenol red. To determine the most effective dose of the *ninl*, *dzank1* and *dync1h1* MOs, 1 nl of diluted MO (containing 2,4,6,8 and 10 ng) was injected into the yolk of one- to two-cell-stage embryos using a Pneumatic Picopump pv280 (World Precision Instruments). A minimum sample size of 50 larvae was used for every condition. After injection, embryos were cultured in E3 embryo medium (5 mM NaCl, 0.17 mM KCl, 0.33 mM CaCl_2_, 0.33 mM MgSO_4_, supplemented with 0.1% methylene blue) at 28°C and subsequently phenotyped at 4 dpf (days post fertilization). Injected embryos were classified into two classes of phenotypes based on the relative severity as compared to age-matched, standard control (5’-cctcttacctcagttacaatttatac-3’; Gene Tools Inc, USA) MO-injected (10 ng) embryos of the same clutch. Images were taken with an Axioplan2 Imaging fluorescence microscope (Zeiss, Germany) equipped with a DC350FX camera (Zeiss, Germany). To determine the efficiency of splice-blocking, RNA was isolated from 50 control MO injected and 50 *dzank1*- (and *ninl*-) splice MO-injected embryos (2 dpf) using the RNeasy mini kit (Qiagen) according to manufacturer’s instructions. Here, 500 ng of total RNA was used to produce first-strand cDNA. Reverse transcription was performed using the Superscript III cDNA synthesis kit (Life Technologies) according to the manufacturer’s instructions. Subsequently, PCR analysis was performed. Primers used for the analysis of *ninl* exon15 morphants are 5’-AAGTATGATGGCCTGGATGC-3’ and 5’-GAGATGTCCTTCCGCTCAAC-3’; primers used for the analysis of *dzank1* exon4 morphants are 5’-GGCAGCACCTCAAATAATCC-3’ and 5’-CTGAAGGTCGATGGCTAAGG-3’; primers used for the analysis of *dzank1* exon8 morphants are 5’-CTCGCTTGACAGCACAAAAC-3’ and 5’-AAAACAGGTCTGGCTTGTCG-3’. Obtained fragments were extracted from a 1% agarose gel using the Nucleospin gel extraction kit (Machery Nagel, USA) and Sanger sequenced. For histological analysis of zebrafish, larvae were fixed in 4% PFA in PBS at 4°C overnight. Embryos were rinsed with PBS and infiltrated in 10% sucrose solution in PBS for two hours. Embryos were positioned (ventral side downwards) in Tissue Tek (Sakura), rapidly frozen in melting isopropyl alcohol and sections (seven μm thickness along the lens/optic nerve axis) were made. Immunohistochemistry was performed using retina sections, derived from four-to-six day old morphants and age-matched control oligo MO-injected zebrafish. The bodipy staining was performed on 5 day old larvae. The sections were washed twice in PBS for 5 minutes, permeabilized with 0.5% triton-x-100 in PBS for two times 10 minutes and followed by three washing steps with PBS for 5 minutes. Sections were then incubated for 10 minutes with bodipy (1:100), DAPI and phalloidin/actin (monoclonal Actin, Abcam lotnr: Ab328–500 (1:400)) diluted in PBS. Subsequent photoreceptor outer segment length measurements were performed blinded as to their injection status (using ImageJ). Equivalent single confocal sections through each eye were selected and the outer segments from 10 adajcent photoreceptors were measured and averaged for each larvae. *P*-values were calculated using Student’s *t*-test (two tailed, unpaired).

### Affinity purifications of protein complexes

HEK293T cells were cultured as described previously [47]. For SILAC experiments, HEK293T cells were grown in SILAC DMEM (PAA), supplemented with 3 mM L-Glutamine (PAA), 10% dialyzed fetal bovine serum (PAA), 0.55 mM lysine and 0.4 mM arginine. Light SILAC medium was supplemented with ^12^C_6_, ^14^N_2_ lysine and ^12^C_6_, ^14^N_4_ arginine. Heavy SILAC medium was supplemented with either ^13^C_6_ lysine and ^13^C_6_, ^15^N_4_ arginine or ^13^C_6_, ^15^N_2_ lysine and ^13^C_6_, ^15^N_4_ arginine. 0.5 mM proline was added to all SILAC media to prevent arginine to proline conversion. All amino acids were purchased from Silantes. For DNA transfections, HEK293T cells were seeded, grown overnight, and then transfected using PEI.

### Tandem affinity purification

HEK293T (human embryonic kidney, ATCC) cells were transfected for 48 hours with either SF-TAP-NINL, SF-TAP-DZANK1, using polyethyleneimine (PEI, Polysciences) as a transfection reagent. Following transfection, cells were lysed in lysis buffer containing 30 mM Tris–HCl (pH 7.4), 150 mM NaCl, 0.5% Nonidet-P40 (NP40), freshly supplemented with protease inhibitor cocktail (Roche), phosphatase inhibitor cocktail II and III (Sigma), for 20 minutes at 4°C. The Streptavidin- and FLAG-based tandem affinity purification steps were performed as previously described [47, 48]. 5% of the final eluate was evaluated by SDS-PAGE followed by silver staining, according to standard protocols, while the remaining 95% were subjected to protein precipitation with chloroform and methanol. Protein precipitates were subsequently subjected to mass spectrometry analysis and peptide identification as previously described [25].

### One-step Strep affinity purification

For SILAC experiments, one step Strep purifications of SF-TAP-tagged proteins and associated protein complexes was performed as described earlier [49]. HEK293T cells, transiently expressing the SF-TAP-tagged constructs were lysed in lysis buffer, containing 0.5% Nonidet-P40, protease inhibitor cocktail (Roche) and phosphatase inhibitor cocktails II and III (Sigma-Aldrich) in TBS (30 mM Tris-HCl (pH 7.4), 150 mM NaCl), for 20 minutes at 4°C. After sedimentation of nuclei at 10,000 x g for 10 minutes, the protein concentration was determined by a Bradford assay, before equal amounts of each lysate were transferred to Strep-Tactin-Superflow beads (IBA) and were incubated for one hour at 4°C on an end-over-end shaker. Then, the resin was washed three times with wash buffer (TBS containing 0.1% NP-40, phosphatase inhibitor cocktail II and III). The protein complexes were eluted by incubation for 10 minutes in Strep-elution buffer (IBA). The eluted samples were concentrated using 10 kDa cut-off VivaSpin 500 centrifugal devices (Sartorius Stedim Biotech) and pre-fractionated using 1D-SDS-Page. Afterwards, the samples were subjected to in-gel tryptic cleavage as described elsewhere [50].

### Protein complex destabilization

For EPASIS, SF-TAP-tagged NINL was over-expressed in HEK293T cells as described above. After 48 hours, cells were lysed as described for SF-TAP analysis and the cleared lysates were incubated with anti-FLAG-M2 agarose resin for 1h. After three washes with wash buffer (TBS containing 0.1% Tergitol-type NP-40 and phosphatase inhibitor cocktails II and III, Sigma-Aldrich), the resin was incubated at 4°C for three minutes with each concentration of SDS (0.001%, 0.005%, 0.01% and 0.02%) in SDS-elution buffer (TBS containing phosphatase inhibitor cocktails II and III). Afterwards, a final elution step with FLAG peptide (200 μg/ml; Sigma-Aldrich) in wash buffer was performed. After every elution step a single wash step was performed. The flow-through was collected and precipitated by methanol-chloroform, before being analyzed by LC-MS/MS.

### Mass spectrometry and data analysis

LC-MS/MS analysis was performed on an Ultimate3000 nano RSLC system (Thermo Scientific) coupled to a LTQ Orbitrap Velos or to an LTQ OrbitrapXL mass spectrometer (Thermo Scientific) by a nano spray ion source. Tryptic peptide mixtures were automatically injected and loaded at a flow rate of 6 μl/min in 0.1% trifluoroacetic acid in HPLC-grade water onto a nano trap column (75 μm internal diameter (i.d.) × 2 cm, packed with Acclaim PepMap100 C18, 3 μm, 100 Å; Thermo Scientific). After five minutes, peptides were eluted and separated on the analytical column (75 μm i.d. × 25 cm, Acclaim PepMap RSLC C18, 2μm, 100 Å; Thermo Scientific) by a linear gradient from 2% to 35% of buffer B (80% acteto-nitrile and 0.08% formic acid in HPLC-grade water) in buffer A (2% aceto-nitrile and 0.1% formic acid in HPLC-grade water) at a flow rate of 300 nl/min over 33 minutes for EPASIS samples, respectively over 80 minutes for SF-TAP and SILAC samples. Remaining peptides were eluted by a short gradient from 35% to 95% buffer B in 5 minutes. The eluted peptides were analyzed by a LTQ Orbitrap Velos or a LTQ Orbitrap XL mass spectrometer. From the high resolution MS pre-scan with a mass range of 300 to 1500, the ten most intense peptide ions were selected for fragment analysis in the linear ion trap if they exceeded an intensity of at least 200 counts and if they were at least doubly charged. The normalized collision energy for CID was set to a value of 35% and the resulting fragments were detected with normal resolution in the linear ion trap. The lock mass option was activated; the background signal with a mass of 445.12002 as lock mass. Every ion selected for fragmentation, was excluded for 20 seconds by dynamic exclusion. Non-quantitative MS/MS data were analyzed, using Mascot (version 2.4, Matrix Science, Boston, MA, USA). Mascot was set up to search the human subset of the Swiss Prot database (Release 2013_12, 20274 entries), assuming trypsin as the digestion enzyme. Mascot was searched with a fragment ion mass tolerance of 0.5 Da and a parent ion tolerance of 10.0 PPM. Oxidation of methionine and was specified as variable modification, iodoacetamide derivative of cysteine as fixed. The Mascot results were loaded in Scaffold (version Scaffold_4, Proteome Software Inc., Portland, OR) to validate MS/MS based peptide and protein identifications. Peptide identifications were accepted if they could be established at greater than 95.0% probability as specified by the Peptide Prophet algorithm [51]. Protein identifications were accepted if they could be established at greater than 99.0% probability and contained at least two identified peptides. Protein probabilities were assigned by the Protein Prophet algorithm [52]. Proteins, which contained similar peptides and could not be differentiated based on MS/MS analysis alone, were grouped to satisfy the principles of parsimony.

For SILAC experiments, all acquired spectra were processed and analyzed, using the MaxQuant software [53] (version 1.3.0.5) and the human subset of the human proteome reference set, provided by SwissProt (release 2012_01 534,242 entries) was used for peptide and protein identification. Cysteine carbamidomethylation was selected as fixed modification, methionine oxidation and protein acetylation were allowed as variable modifications. The peptide and protein false discovery rates were set to 1%. Contaminants like keratins were removed. Proteins, identified and quantified by at least two unique peptides were considered for further analysis. The significance values were determined by Perseus tool (part of MaxQuant), using significance A. Label-free quantification and statistical analysis of the EPASIS data were performed as previously described [25] using MaxQuant (version 1.3.0.5). The human subset of the human proteome reference set, provided by SwissProt (Release 2012_01 534,242 entries) was used for peptide and protein identification. Seven biological replicates with five fractions each were performed for the NINL experiment and three biological replicates for the control experiment, resulting in a total number of 50 individual samples being measured. Proteins had to be present in at least 4/7 (57%) repeated experiments to be considered for further analysis. The reproducibility of the experiments was analyzed as already described [25] and is shown in S6 and S7 Figs To assign the proteins to the pre-defined sub-complexes (S4 Table [26, 54]), the EPD threshold was determined by a stepwise parameter search (n = 1000), which resulted in a value of 0.089 (S8 Fig).

### Transmission electron microscopy (TEM)

Zebrafish (4 dpf) were fixed at 4°C overnight in a freshly prepared mixture of 2,5% glutaraldehyde and 2% PFA in 0.1 M sodium cacodylate buffer (pH 7.4). After rinsing in buffer, specimens were post-fixated in a freshly prepared mixture, containing 1% osmium tetroxide and 1% potassium ferrocyanide in 0.1 M sodium cacodylate buffer (pH 7.4), at room temperature during 2 hours. After rinsing, tissues were dehydrated through a graded series of ethanol and embedded in epon. Ultrathin (rostrocaudally) sections (70 nm), comprising zebrafish eyes at the optic nerve level, were collected on Formvar coated grids, subsequently stained with 2% uranyl acetate and Reynold’s lead citrate, and examined with a Jeol1010 electron microscope. Using Adobe Photoshop version 8.0, TEM images were adjusted for brightness and contrast. To compare the degree of vesiculation in the inner segments of the various experimental groups, quantitative TEM analysis was accomplished. To this end, 8000x magnification images of the central retina (50% middle arc length) were acquired. For each zebrafish group, several eyes and fields of view in the retina were evaluated (S5 Table). For each field of view, the total number of photoreceptor cells was counted. Finally, each photoreceptor cell was evaluated for presence of vesicles. The statistical significance of differences between two groups was assessed using the independent samples Student’s *t*-test (SPSS 20.0). Morphant groups were analyzed and compared versus the wild type group, as well as versus the mock-injected group. The statistical significance was set at *p* < 0.05. Data are presented as means ± SEM.

### Zebrafish OKR assay

The OKR was measured by a previously described method [55]. Zebrafish larvae were mounted in an upright position in 3% methylcellulose in a small Petri dish. The Petri dish was placed on a platform surrounded by a rotating drum 8 cm in diameter. A pattern of alternating black and white vertical stripes was displayed on the drum interior (each stripe subtended an angle of 36°C). Larvae (4 dpf) were visualized through a stereomicroscope positioned over the drum and illuminated with fiberoptic lights. Eye movements were recorded while larvae were optically stimulated by the rotating stripes. Larvae were subjected to a protocol of a 30 seconds counterclockwise rotation, a 10 seconds rest, and a 30 seconds clockwise rotation.

### Melanosome transport assay

To induce melanosome retraction P5 larvae were exposed to epinephrine (Sigma E4375) at a final concentration of 500 μg/ml. Melanosome retraction was continuously monitored under the microscope and the endpoint was scored when all melanosomes in the head (and the trunk) were perinuclear [28]. *P*-values were obtained using Student’s *t*-test (two tailed, unpaired).

### Statistical analyses

For all quantifications of zebrafish experiments, the Graphpad Prism6 software (http://www.graphpad.com/scientific-software/prism/) was employed to generate scatter plots, calculate mean values and SEM values, and perform statistical tests. Continuous data was analyzed using two-tailed, unpaired Student’s t-test and categorical data was analyzed using Fisher’s exact test.

## Supporting Information

S1 Fig Specificity of the *dzank1* ex8 spMO. Titration curve of the dzank1 ex8 spMO scored on ambulatory activity shows an increased incidence of the phenotype with an increasing dose (a). (b) Co-injection of 6 ng *dzank1* ex8 spMO with 150 pg capped MO-resistant mRNA encoding human DZANK1 reduced the incidence of phenotypes including ambulatory activity, small eyes (c, d, e) (n>95/group, p<0.0001 (two-tailed Fisher’s exact test) and restored photoreceptor outer segment lengths (n = 13, P<0.001 (two-tailed, unpaired Student’s *t*-test), c’, d’, e’, f). (f) Quantification of photoreceptor outer segment lengths revealed a significant increase in length in the *DZANK1* rescue group (3.0+/-0.4 μm) as compared to *dzank1* morphants (6ng/nl; 1.9+/-0.25 μm) (*P*<0.001; two-tailed, unpaired Student’s *t*-test). Bars indicate mean OS length per group and Standard error of the mean (SEM) (g) Characterization of the effect of the *dzank1* ex8 spMO at 2 and 4 dpf by RT-PCR analysis. Injection of various amounts of MO resulted in the (partial) retention of intron7, leading to a premature termination of translation. PCR fragments were analyzed by Sanger sequencing. Scale bars represent 500 μm (c-e) and 15 μm (c’-e’).
(TIF)

S2 Fig Recapitulation of the phenotype by a second *dzank1* spMO targeting exon4. Injection of a *dzank1* ex4 spMO-injected larvae completely recapitulates the phenotype observed in *dzank1* ex8 spMO-injected larvae, including pericardial edema, small eyes, defects in ambulatory activity (b) and shortened photoreceptor outer segments (b’) as compared to control MO-injected larvae from the same clutch (a,a’). (c) Characterization of the effect of the *dzank1* ex4 spMO at 2 dpf by non-quantitative RT-PCR analysis. Injection of various amounts of MO resulted in the (partial) skipping of exon4, leading to a premature termination of translation. PCR fragments were analyzed by Sanger sequencing. Scale bars represent 500 μm (a-b) and 15 μm (a’-b’).
(TIF)

S3 Fig DZANK1 co-localizes with DYNLL1 at the base of the cilia. (a-c) eCFP-DZANK1 (green signal) and mRFP-DYNLL1 (red signal) localized to both centrioles of the centrosome and to the basal body of the cilia marked by GT335 (Cyanid signal). After co-expression, both proteins localized at the basal body of the cilia at the centrosome. c; yellow signal). Nuclei are stained with DAPI (blue signal). (d) Co-immunoprecipitation of DZANK1 FL with DYNLL1, but not with LRRK2. The immunoblot (IB) in the top panel shows that HA-tagged DYNLL1 co-immunoprecipitated with Strep/FLAG-tagged DZANK1 (lane 2), whereas unrelated FLAG-tagged LRRK2 (lane 3) did not. The anti-HA immunoprecipitates are shown in the middle panel; protein input is shown in the bottom panel. (d’) Reciprocal IP experiments using anti-FLAG antibodies confirmed the co-immunoprecipitation of HA-tagged DYNLL1 with Strep/FLAG-tagged DZANK1 (lane 2) and not with LRRK2 (lane 3) shown in the top panel. The anti-FLAG immunoprecipitates are shown in the middle panel; protein input is shown in the bottom panel. Scale bars represent 10 μm (a-c).
(TIF)

S4 Fig DZANK1 co-localizes with DYNLL2 at the base of the cilia. (a-c) eCFP DZANK1 (a; green signal) and mRFP-DYNLL2 (b; red signal) co-localizes at the basal body of the cilia in the centrosome (c; yellow signal). Nuclei were stained with DAPI (blue signal). (d-d’) Co-immunoprecipitation of DZANK1 FL with DYNLL2, but not with LRRK2. The immunoblot (IB) in the top panel shows that 3xHA-tagged DYNLL2 co-immunoprecipitates with Strep/FLAG-tagged DZANK1 (lane 2), whereas unrelated FLAG-tagged LRRK2 (lane 3) does not. The anti-HA immunoprecipitates are shown in the middle panel; protein input is shown in the bottom panel. (d’) Reciprocal IP experiments using anti-FLAG antibodies confirm the co-immunoprecipitation of 3xHA-tagged DYNLL2 with Strep/FLAG-tagged DZANK1 (lane 2) but not with LRRK2 (lane 3) shown in the top panel. The anti-FLAG immunoprecipitations are shown in the middle panel; protein input is shown in the bottom panel. Scale bars represent 10 μm (a-c).
(TIF)

S5 Fig EPASIS of the NINL protein complex. (a)Visualization of the elution profiles of the known consensus protein groups, dynactin (DCTN, red), cytoplasmic dynein 1 module (DYN, blue), after analysis by liquid chromatography coupled to tandem mass spectrometry (LC-MS/MS) and label-free quantification. On the y-axis the cumulative relative abundance is plotted against the stepwise increasing SDS concentration on the x-axis. (b) Nonmetric multidimensional scaling ordination plot based on the Euclidean distances of elution profiles (stress 0.04). Data points (n = 86) present the average of replicated data (n = 7). (c) Sub-module organization of the NINL interactome, showing its putative sub-structure as determined by EPASIS. The respective modules are highlighted by colored clouds, the known members of the sub-modules are shown in full color, the new members in grey. Additionally to the known members of the dynactin module, several, potentially new candidates could be assigned to the dynactin module (ACTR10, RBM14, BIRC6, SMC4, MARK2, DNAJA1, CEP170, ATAD3A and PRPF19) with an Elution Profile Distance (EPD) ≤ 0.077. The second sub-module consists of proteins from the cytoplasmic dynein 1 motor complex and eluted between a SDS concentration of 0.001 and 0.01% from the NINL protein complex. Four further proteins were determined as potential new candidates to this module (MRPS27, ACAD11, PAFAH1B1 and CLIP1; EPD ≤ 0.015). Interestingly, the dynactin “pointed-end complex” protein DCTN5 was in our experiments clearly assigned to the dynein module. The distance of the modules from the bait, along the curved, dashed line, reflects the resistance of the interaction to SDS and correlates with stability of association.
(TIF)

S6 Fig Reproducibility of the NINL EPASIS. Scatter plots of log2-transformed protein (n = 86) intensities from replicated experiments (experiment 1–7). Orthogonal regression lines are shown in red; Pearson correlation coefficients (r) and their 95% confidence intervals (ci) are shown.
(TIF)

S7 Fig Correlation matrix plot of the NINL EPASIS. Correlation matrix plot of log2-transformed protein (n = 86) intensities for all concentration steps. Correlation scores of Spearman’s test statistic are displayed and color-coded.
(TIF)

S8 Fig Threshold estimation for the elution profile distance (EPD) of the NINL EPASIS. For stepwise increasing thresholds (n = 1000), the specificity (black line) and sensitivity (blue line) to detect known consensus profile members are displayed. The grey line represents the selected threshold of 0.089 leading to the selection of 13 candidate proteins and 13 reference group proteins.
(TIF)

S9 Fig *Ninl* or *Dzank1* knockdown in zebrafish leads to mis-localization of rhodopsin. Immunofluorescence with anti-opsin antibody 4D2 demonstrated mis-localization of opsins (indicated by arrows) in the photoreceptor cell body in *ninl* (b-b’) and *dzank1* (c-c’) morphants, compared to controls, where opsins are restricted to the outer segments (a-a’). (a’-c’) are the white boxed areas of (a-c). Larvae are 4 dpf. Scale bars represent 50 μm (a-c) and 15 μm (a’-c’).
(TIF)

S1 Table GST pull-down from retinal extracts. GST pull-down analysis from bovine retinal extracts with N-terminally GST-fused NINLisoB_aa538-825 (n = 4) or GST alone (n = 3) as a control. Shown are the number of unique peptides that were identified for each protein detected by mass spectrometry. Proteins identified in less than three out of four experiments and/or in GST-control experiments were removed.
(XLS)

S2 Table TAP-data and SILAC data. SF-TAP analysis with over-expressed N-terminally SF-TAP-tagged proteins in HEK293T cells. Shown are the number of unique identified peptides as well as the sequence coverage for each protein detected by mass spectrometry. Proteins identified in the SF-TAP analysis of empty vector control experiments were removed. SILAC analysis with over-expressed N-terminally SF-TAP-tagged proteins in HEK293T cells. Shown are the ratios and significance value for WT/SF-control experiments.
(XLSX)

S3 Table EPD-values for EPASIS of the NINL protein complex. EPD to consensus profiles were calculated for each protein displayed. Proteins with an EPD equal or less than 0.089 were assigned to a consensus profile. DCTN: dynactin module, DCTN_cand: proteins with EPD-value in the range of DCTN, DYN: Cytoplasmic dynein 1 module, DYN_cand: proteins with EPD-value in the range of DYN, unknown: unassigned proteins.
(XLSX)

S4 Table Consensus protein groups NINL interactome for EPASIS. In the first column the corresponding sub-modules are depicted, the second column indicates the Uniprot accession number, in the third column the gene names are shown and the last column indicated the reference for the module association. The assignment to the different modules was done according to literature and by interpretation of the SF-TAP and SILAC data.
(DOC)

S5 Table Specimens (central retina) analyzed for ultrastructural TEM analysis. (DOC)
