# Supplementary figures and images for: NINL and DZANK1 Co-function in Vesicle Transport and Are Essential for Photoreceptor Development in Zebrafish
Source: PLoS Genet. 2015 Oct 20;11(10):e1005574. doi: 10.1371/journal.pgen.1005574 (PMC4617706; doi:10.1371/journal.pgen.1005574)

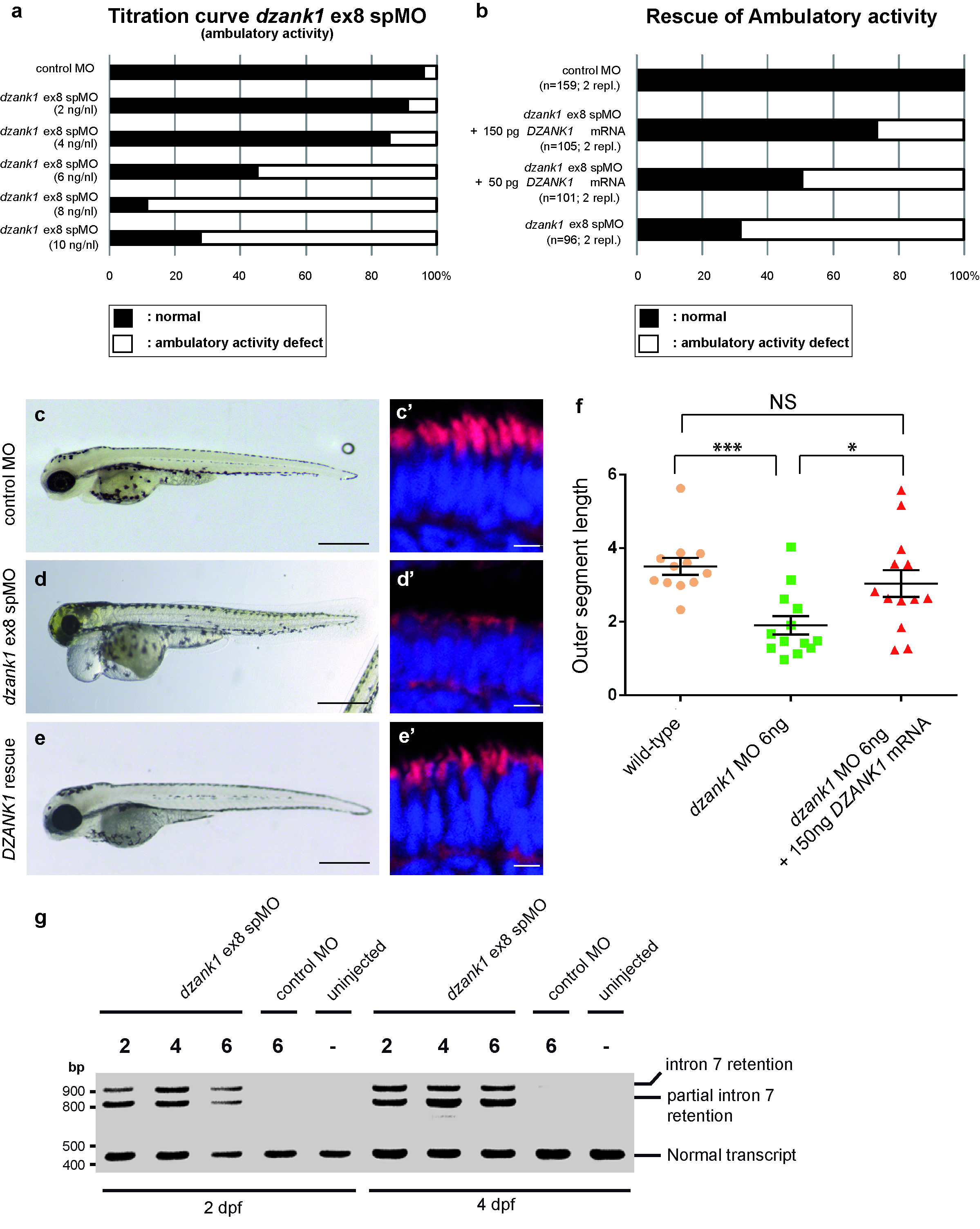

Supplement: S1 Fig — Titration curve of the dzank1 ex8 spMO scored on ambulatory activity shows an increased incidence of the phenotype with an increasing dose (a). (b) Co-injection of 6 ng dzank1 ex8 spMO with 150 pg capped MO-resistant mRNA encoding human DZANK1 reduced the incidence of phenotypes including ambulatory activity, small eyes (c, d, e) (n>95/group, p<0.0001 (two-tailed Fisher’s exact test) and restored photoreceptor outer segment lengths (n = 13, P<0.001 (two-tailed, unpaired Student’s t-test), c’, d’, e’, f). (f) Quantification of photoreceptor outer segment lengths revealed a significant increase in length in the DZANK1 rescue group (3.0+/-0.4 μm) as compared to dzank1 morphants (6ng/nl; 1.9+/-0.25 μm) (P<0.001; two-tailed, unpaired Student’s t-test). Bars indicate mean OS length per group and Standard error of the mean (SEM) (g) Characterization of the effect of the dzank1 ex8 spMO at 2 and 4 dpf by RT-PCR analysis. Injection of various amounts of MO resulted in the (partial) retention of intron7, leading to a premature termination of translation. PCR fragments were analyzed by Sanger sequencing. Scale bars represent 500 μm (c-e) and 15 μm (c’-e’). (TIF) [file pgen.1005574.s001.tif]

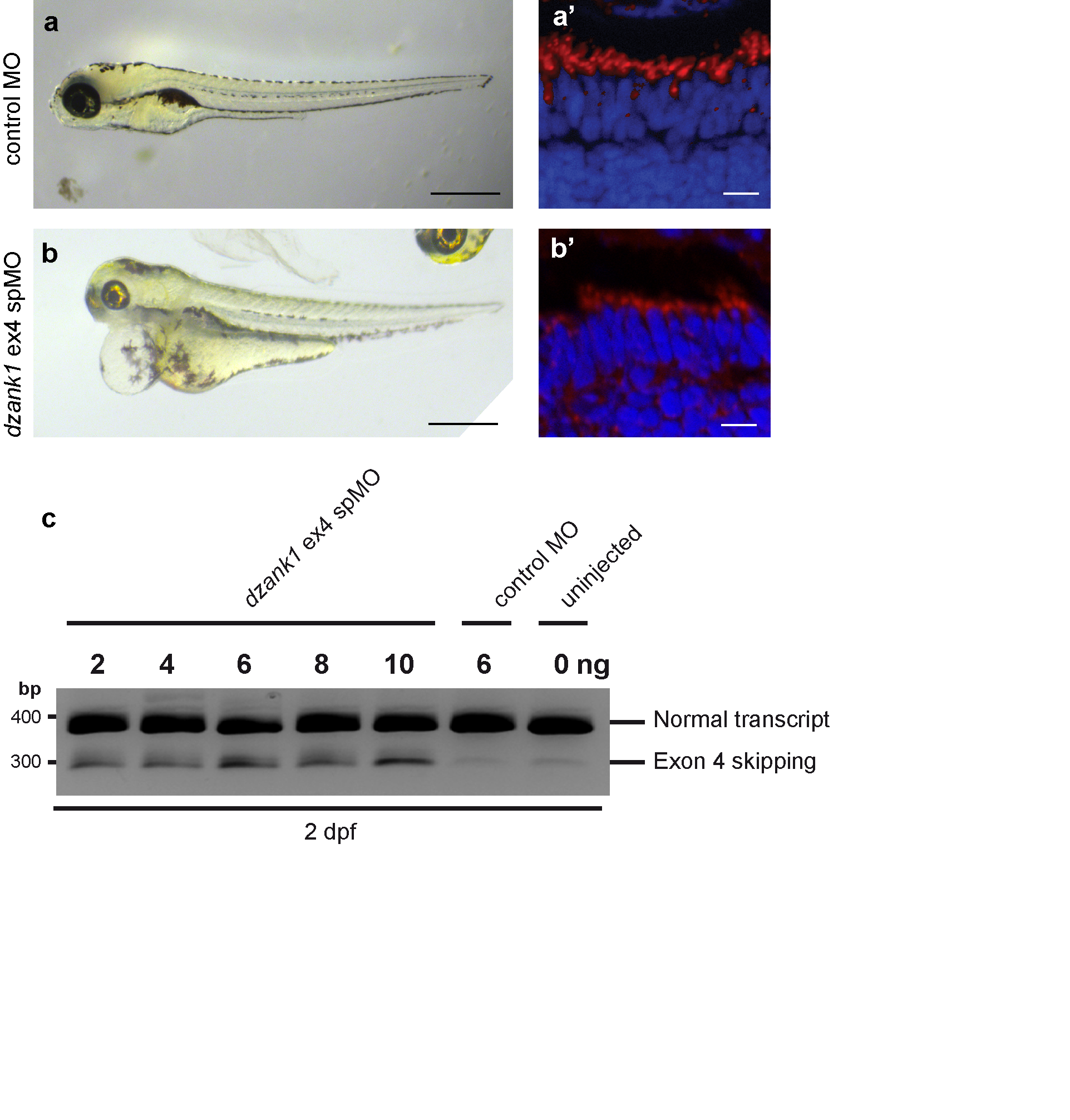

Supplement: S2 Fig — Injection of a dzank1 ex4 spMO-injected larvae completely recapitulates the phenotype observed in dzank1 ex8 spMO-injected larvae, including pericardial edema, small eyes, defects in ambulatory activity (b) and shortened photoreceptor outer segments (b’) as compared to control MO-injected larvae from the same clutch (a,a’). (c) Characterization of the effect of the dzank1 ex4 spMO at 2 dpf by non-quantitative RT-PCR analysis. Injection of various amounts of MO resulted in the (partial) skipping of exon4, leading to a premature termination of translation. PCR fragments were analyzed by Sanger sequencing. Scale bars represent 500 μm (a-b) and 15 μm (a’-b’). (TIF) [file pgen.1005574.s002.tif]

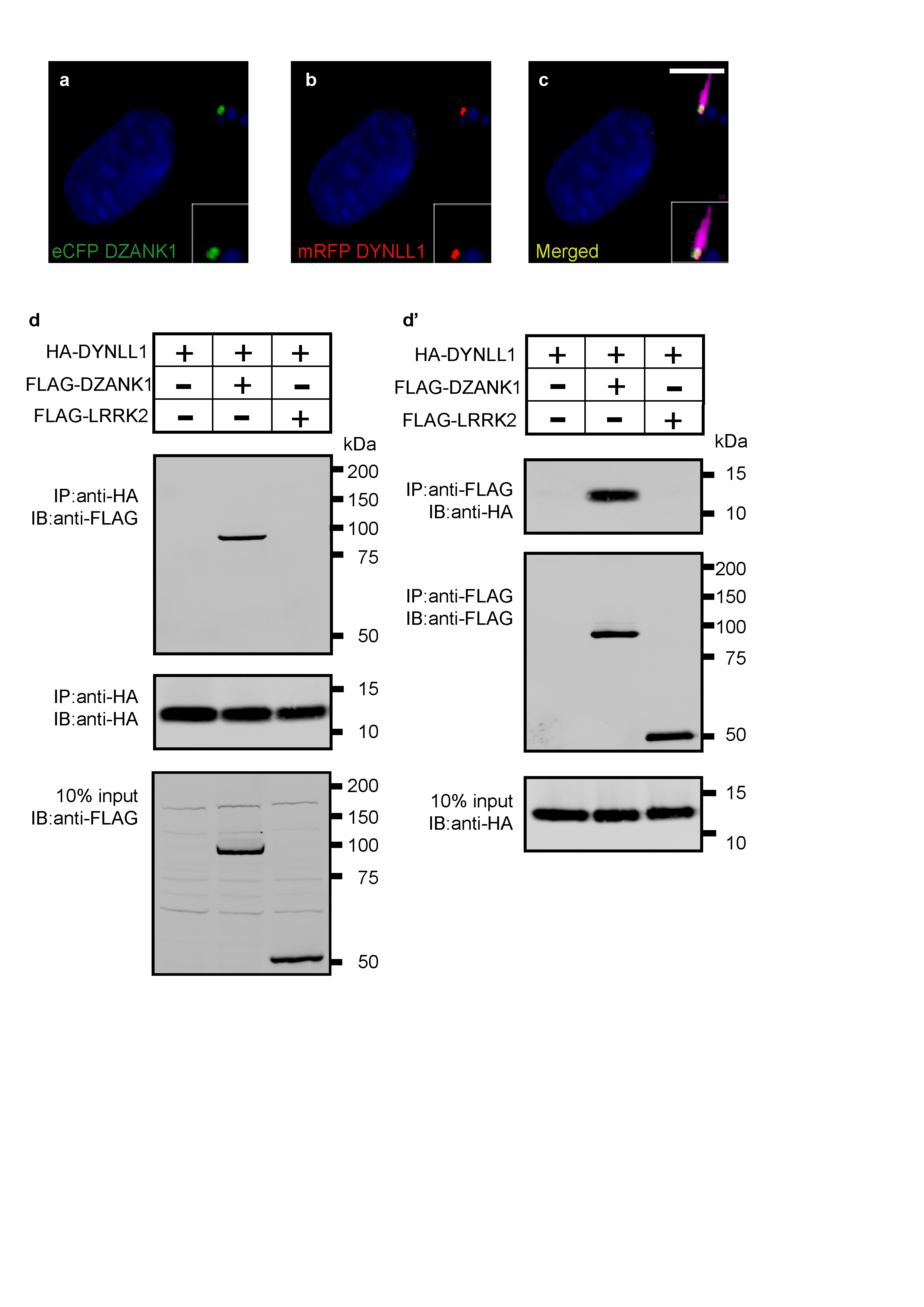

Supplement: S3 Fig — (a-c) eCFP-DZANK1 (green signal) and mRFP-DYNLL1 (red signal) localized to both centrioles of the centrosome and to the basal body of the cilia marked by GT335 (Cyanid signal). After co-expression, both proteins localized at the basal body of the cilia at the centrosome. c; yellow signal). Nuclei are stained with DAPI (blue signal). (d) Co-immunoprecipitation of DZANK1 FL with DYNLL1, but not with LRRK2. The immunoblot (IB) in the top panel shows that HA-tagged DYNLL1 co-immunoprecipitated with Strep/FLAG-tagged DZANK1 (lane 2), whereas unrelated FLAG-tagged LRRK2 (lane 3) did not. The anti-HA immunoprecipitates are shown in the middle panel; protein input is shown in the bottom panel. (d’) Reciprocal IP experiments using anti-FLAG antibodies confirmed the co-immunoprecipitation of HA-tagged DYNLL1 with Strep/FLAG-tagged DZANK1 (lane 2) and not with LRRK2 (lane 3) shown in the top panel. The anti-FLAG immunoprecipitates are shown in the middle panel; protein input is shown in the bottom panel. Scale bars represent 10 μm (a-c). (TIF) [file pgen.1005574.s003.tif]

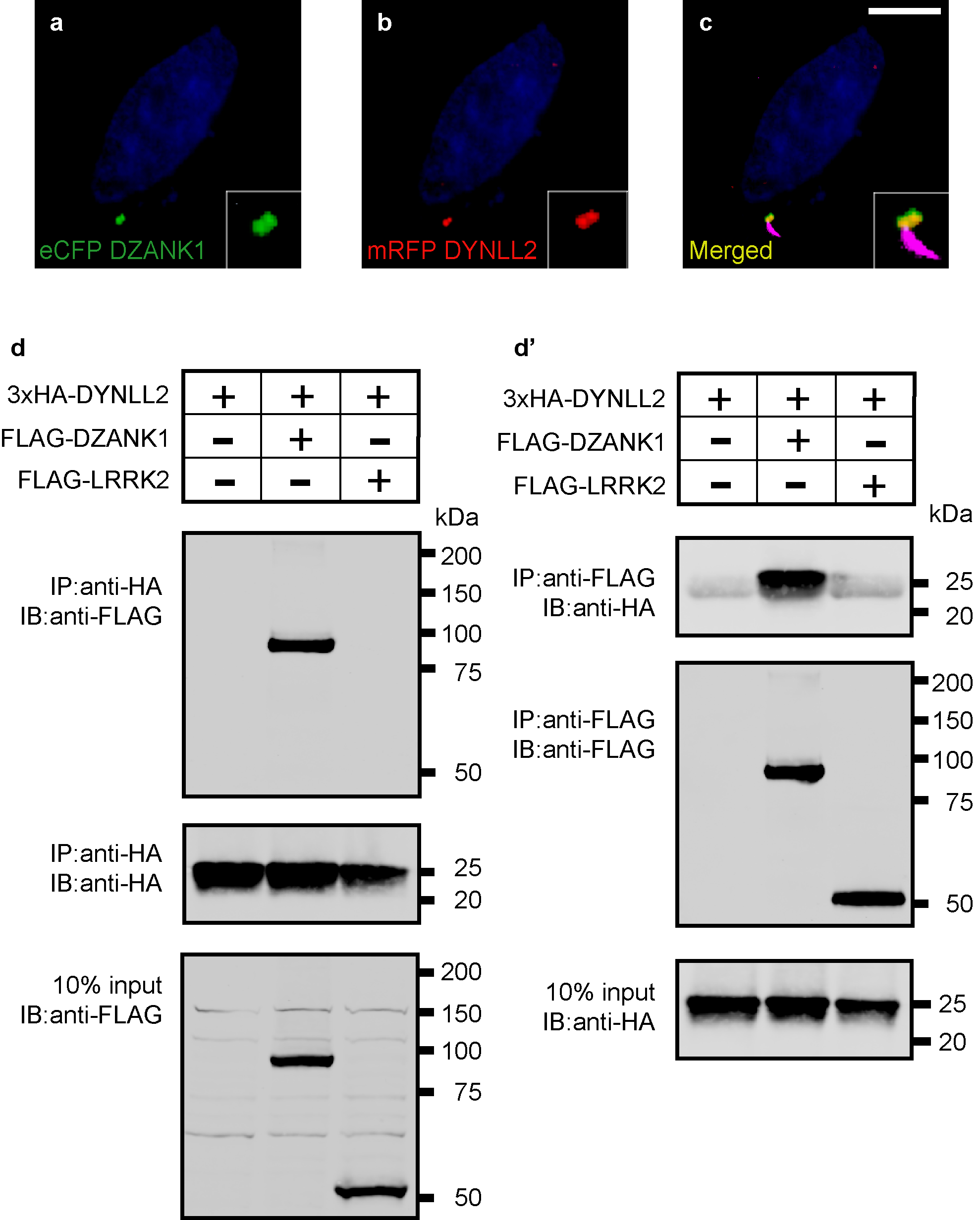

Supplement: S4 Fig — (a-c) eCFP DZANK1 (a; green signal) and mRFP-DYNLL2 (b; red signal) co-localizes at the basal body of the cilia in the centrosome (c; yellow signal). Nuclei were stained with DAPI (blue signal). (d-d’) Co-immunoprecipitation of DZANK1 FL with DYNLL2, but not with LRRK2. The immunoblot (IB) in the top panel shows that 3xHA-tagged DYNLL2 co-immunoprecipitates with Strep/FLAG-tagged DZANK1 (lane 2), whereas unrelated FLAG-tagged LRRK2 (lane 3) does not. The anti-HA immunoprecipitates are shown in the middle panel; protein input is shown in the bottom panel. (d’) Reciprocal IP experiments using anti-FLAG antibodies confirm the co-immunoprecipitation of 3xHA-tagged DYNLL2 with Strep/FLAG-tagged DZANK1 (lane 2) but not with LRRK2 (lane 3) shown in the top panel. The anti-FLAG immunoprecipitations are shown in the middle panel; protein input is shown in the bottom panel. Scale bars represent 10 μm (a-c). (TIF) [file pgen.1005574.s004.tif]

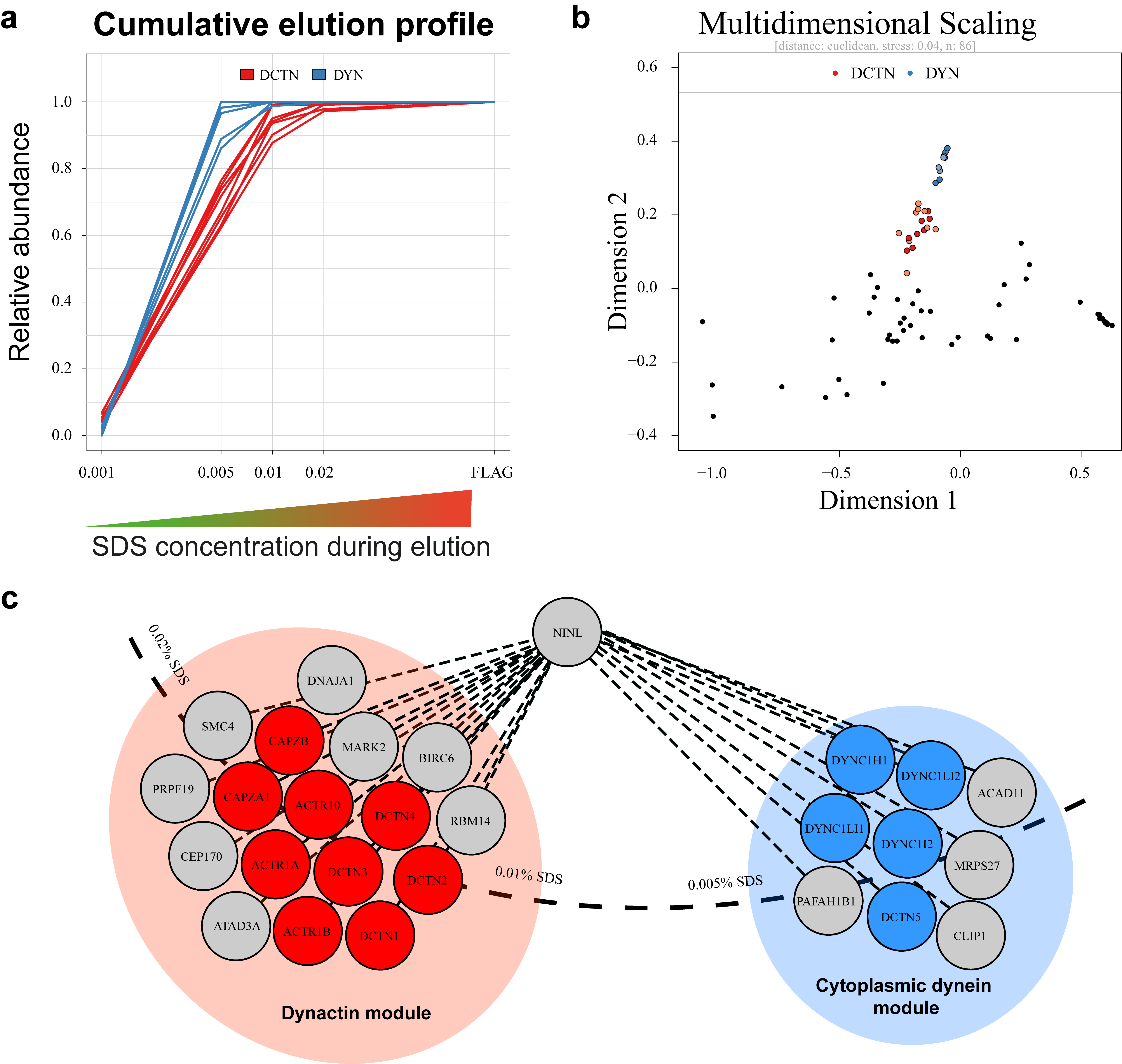

Supplement: S5 Fig — (a)Visualization of the elution profiles of the known consensus protein groups, dynactin (DCTN, red), cytoplasmic dynein 1 module (DYN, blue), after analysis by liquid chromatography coupled to tandem mass spectrometry (LC-MS/MS) and label-free quantification. On the y-axis the cumulative relative abundance is plotted against the stepwise increasing SDS concentration on the x-axis. (b) Nonmetric multidimensional scaling ordination plot based on the Euclidean distances of elution profiles (stress 0.04). Data points (n = 86) present the average of replicated data (n = 7). (c) Sub-module organization of the NINL interactome, showing its putative sub-structure as determined by EPASIS. The respective modules are highlighted by colored clouds, the known members of the sub-modules are shown in full color, the new members in grey. Additionally to the known members of the dynactin module, several, potentially new candidates could be assigned to the dynactin module (ACTR10, RBM14, BIRC6, SMC4, MARK2, DNAJA1, CEP170, ATAD3A and PRPF19) with an Elution Profile Distance (EPD) ≤ 0.077. The second sub-module consists of proteins from the cytoplasmic dynein 1 motor complex and eluted between a SDS concentration of 0.001 and 0.01% from the NINL protein complex. Four further proteins were determined as potential new candidates to this module (MRPS27, ACAD11, PAFAH1B1 and CLIP1; EPD ≤ 0.015). Interestingly, the dynactin “pointed-end complex” protein DCTN5 was in our experiments clearly assigned to the dynein module. The distance of the modules from the bait, along the curved, dashed line, reflects the resistance of the interaction to SDS and correlates with stability of association. (TIF) [file pgen.1005574.s005.tif]

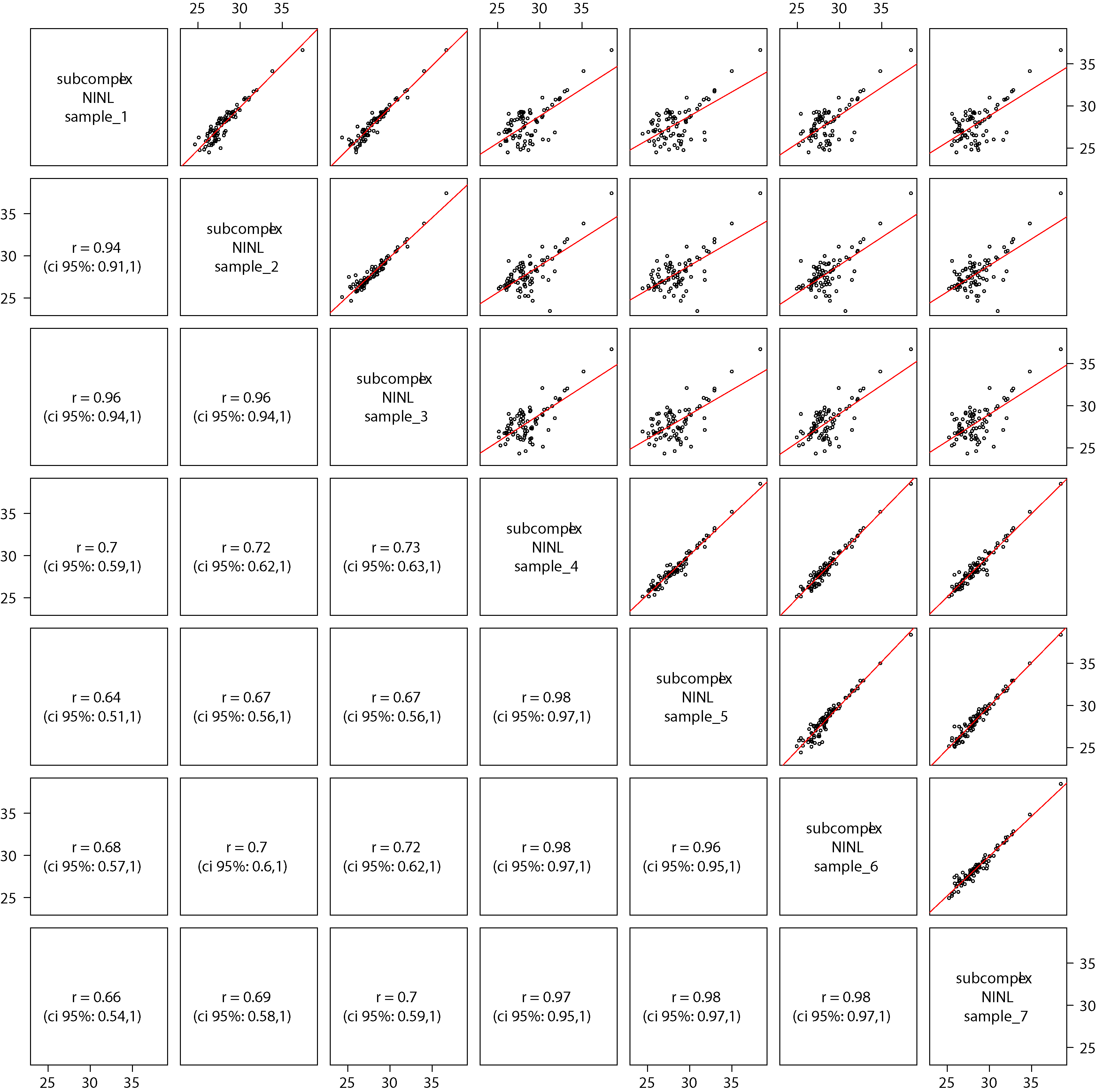

Supplement: S6 Fig — Scatter plots of log2-transformed protein (n = 86) intensities from replicated experiments (experiment 1–7). Orthogonal regression lines are shown in red; Pearson correlation coefficients (r) and their 95% confidence intervals (ci) are shown. (TIF) [file pgen.1005574.s006.tif]

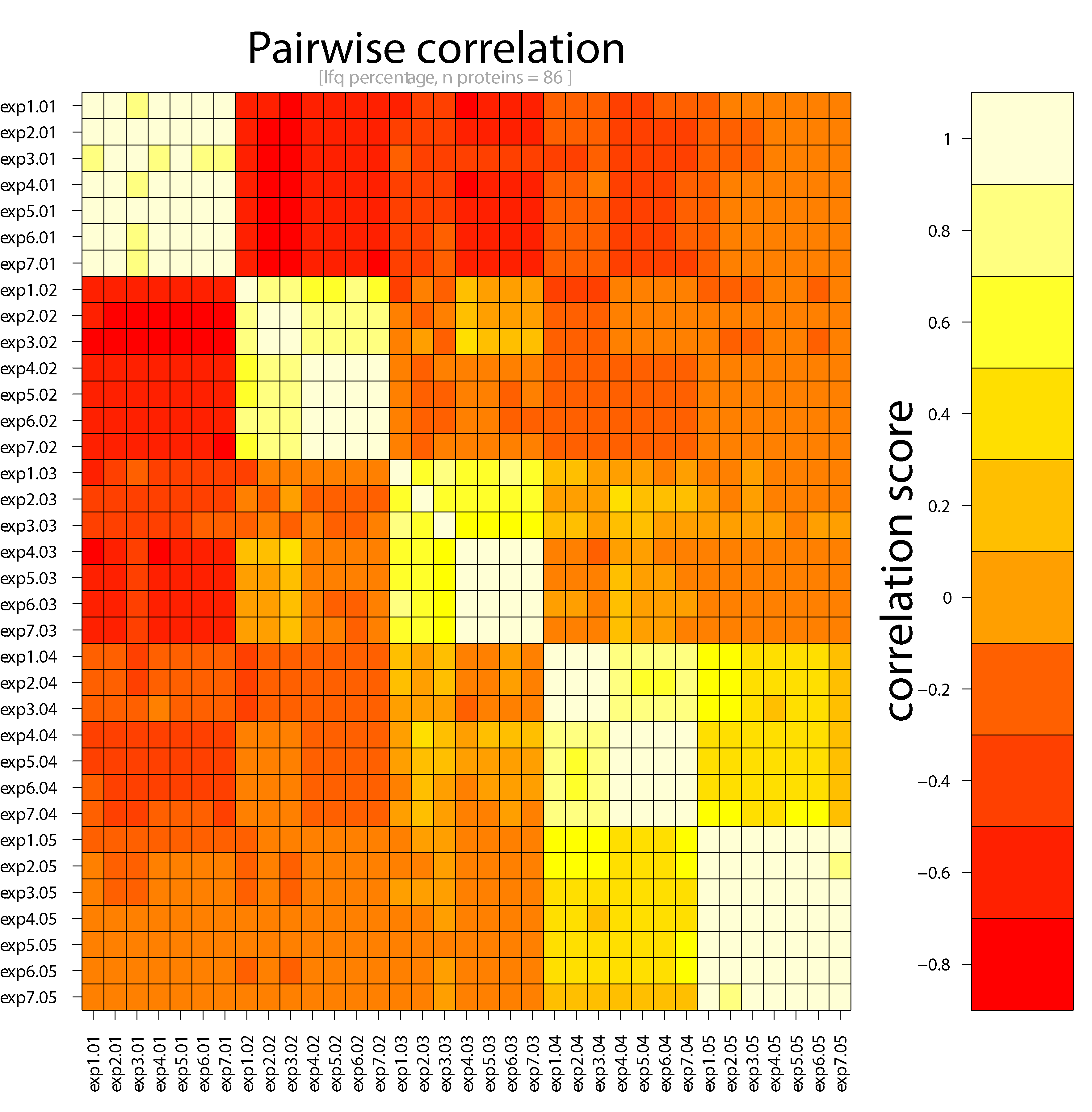

Supplement: S7 Fig — Correlation matrix plot of log2-transformed protein (n = 86) intensities for all concentration steps. Correlation scores of Spearman’s test statistic are displayed and color-coded. (TIF) [file pgen.1005574.s007.tif]

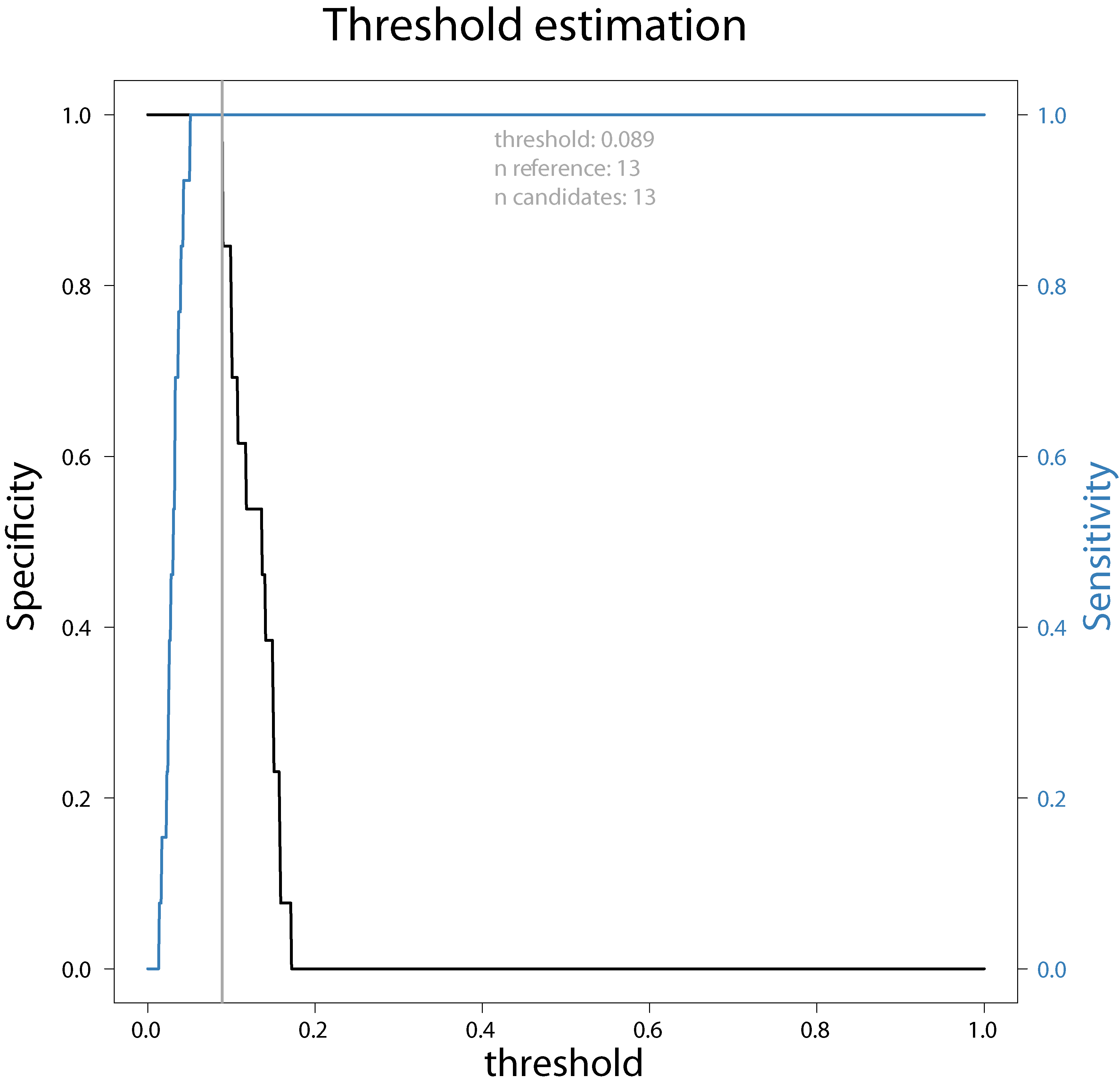

Supplement: S8 Fig — For stepwise increasing thresholds (n = 1000), the specificity (black line) and sensitivity (blue line) to detect known consensus profile members are displayed. The grey line represents the selected threshold of 0.089 leading to the selection of 13 candidate proteins and 13 reference group proteins. (TIF) [file pgen.1005574.s008.tif]

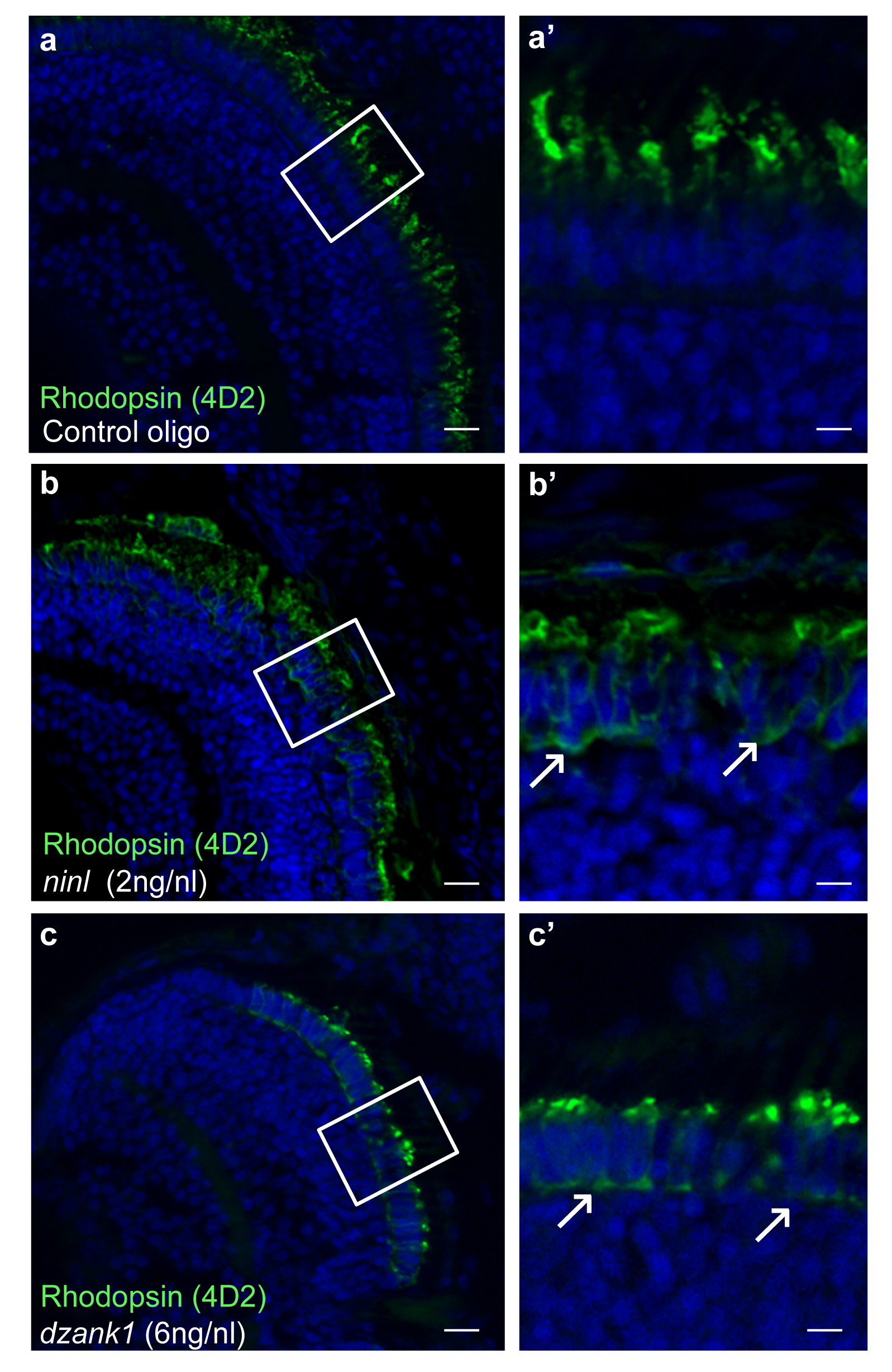

Supplement: S9 Fig — Immunofluorescence with anti-opsin antibody 4D2 demonstrated mis-localization of opsins (indicated by arrows) in the photoreceptor cell body in ninl (b-b’) and dzank1 (c-c’) morphants, compared to controls, where opsins are restricted to the outer segments (a-a’). (a’-c’) are the white boxed areas of (a-c). Larvae are 4 dpf. Scale bars represent 50 μm (a-c) and 15 μm (a’-c’). (TIF) [file pgen.1005574.s009.tif]
