# Supplementary material for: NINL and DZANK1 Co-function in Vesicle Transport and Are Essential for Photoreceptor Development in Zebrafish
Source: PLoS Genet. 2015 Oct 20;11(10):e1005574. doi: 10.1371/journal.pgen.1005574 (PMC4617706; doi:10.1371/journal.pgen.1005574)
Supplement: S4 Table — In the first column the corresponding sub-modules are depicted, the second column indicates the Uniprot accession number, in the third column the gene names are shown and the last column indicated the reference for the module association. The assignment to the different modules was done according to literature and by interpretation of the SF-TAP and SILAC data. (DOC) [file pgen.1005574.s013.doc]

| **Putative**  **module** | **Uniprot#** | **Gene Name** | **Reference** |
| --- | --- | --- | --- |
| DCTN | Q14203 | DCTN1 | Schroer et al. 2004 |
| DCTN | Q13561 | DCTN2 | Schroer et al. 2004 |
| DCTN | O75935 | DCTN3 | Schroer et al. 2004 |
| DCTN | Q9UJW0 | DCTN4 | Schroer et al. 2004 |
| DCTN | P52907 | CAPZA1 | Schroer et al. 2004 |
| DCTN | P47756 | CAPZB | Schroer et al. 2004 |
| DCTN | P61163 | ACTR1A | Schroer et al. 2004 |
| DCTN | P42025 | ACTR1B | Schroer et al. 2004 |
| DYN | Q14204 | DYNC1H1 | Allan 2011 |
| DYN | Q9Y6G9 | DYNC1LI1 | Allan 2011 |
| DYN | Q13409 | DYNC1I2 | Allan 2011 |
| DYN | O43237 | DYNC1LI2 | Allan 2011 |
| DYN | Q9BTE1 | DCTN5 | Schroer et al. 2004 |
