# Supplementary material for: NINL and DZANK1 Co-function in Vesicle Transport and Are Essential for Photoreceptor Development in Zebrafish
Source: PLoS Genet. 2015 Oct 20;11(10):e1005574. doi: 10.1371/journal.pgen.1005574 (PMC4617706; doi:10.1371/journal.pgen.1005574)
Supplement: S5 Table — (DOC) [file pgen.1005574.s014.doc]

| **Zebrafish Group** | **Eyes (n)** | **Fields of view (n)** | **Photoreceptor cells (n)** |
| --- | --- | --- | --- |
| **Wildtype** | 10 | 21 | 102 |
| **Control Oligo (10ng/nl)** | 8 | 18 | 111 |
| **Ninl (2ng/nl)** | 6 | 18 | 115 |
| **Ninl (0.5ng/nl)** | 8 | 11 | 80 |
| **Dzank1 (6ng/nl)** | 9 | 18 | 139 |
| **Dzank1 (1ng/nl)** | 8 | 12 | 134 |
| **Combi (dzank1 + ninl)**  **(1 ng/nl + 0.5ng/nl)** | 7 | 20 | 142 |
